# Cyclic nucleotide gated channel gene family in tomato: genome-wide identification and functional analyses in disease resistance

**DOI:** 10.3389/fpls.2015.00303

**Published:** 2015-05-05

**Authors:** Mumtaz A. Saand, You-Ping Xu, Wen Li, Ji-Peng Wang, Xin-Zhong Cai

**Affiliations:** ^1^Institute of Biotechnology, College of Agriculture and Biotechnology, Zhejiang UniversityHangzhou, China; ^2^Centre of Analysis and Measurement, Zhejiang UniversityHangzhou, China

**Keywords:** cyclic nucleotide gated channel (CNGC), tomato, genome-wide identification, resistance, PAMP-triggered immunity, *Sclerotinia sclerotiorum*

## Abstract

The cyclic nucleotide gated channel (CNGC) is suggested to be one of the important calcium conducting channels. Nevertheless, genome-wide identification and systemic functional analysis of *CNGC* gene family in crop plant species have not yet been conducted. In this study, we performed genome-wide identification of *CNGC* gene family in the economically important crop tomato (*Solanum lycopersicum* L.) and analyzed function of the group IVb *SlCNGC* genes in disease resistance. Eighteen *CNGC* genes were identified in tomato genome, and four *CNGC* loci that were misannotated at database were corrected by cloning and sequencing. Detailed bioinformatics analyses on gene structure, domain composition and phylogenetic relationship of the *SlCNGC* gene family were conducted and the group-specific feature was revealed. Comprehensive expression analyses demonstrated that *SlCNGC* genes were highly, widely but differently responsive to diverse stimuli. Pharmacological assays showed that the putative CNGC activators cGMP and cAMP enhanced resistance against *Sclerotinia sclerotiorum*. Silencing of group IVb *SlCNGC* genes significantly enhanced resistance to fungal pathogens *Pythium aphanidermatum* and *S. sclerotiorum*, strongly reduced resistance to viral pathogen *Tobacco rattle virus*, while attenuated PAMP- and DAMP-triggered immunity as shown by obvious decrease of the flg22- and AtPep1-elicited hydrogen peroxide accumulation in *SlCNGC*-silenced plants. Additionally, silencing of these *SlCNGC* genes significantly altered expression of a set of Ca^2+^ signaling genes including *SlCaMs*, *SlCDPKs,* and *SlCAMTA3*. Collectively, our results reveal that group IV *SlCNGC* genes regulate a wide range of resistance in tomato probably by affecting Ca^2+^ signaling.

## Introduction

The cyclic nucleotide gated channels (CNGCs) are ligand-gated cation channels localized typically in plasma membrane (Chin et al., 2009; Ma et al., 2009). Plant CNGCs are composed of cytoplasmic C-terminal calmodulin binding (CaMB) domain and cyclic nucleotide-binding (CNB) domain as well as N-terminal hexa-transmembrane (TM) domains. The TM domains form a pore to facilitate cation transport (Zelman et al., 2012). Individual plant *CNGC* genes were identified in several plant species including barley (Schuurink et al., 1998), *Arabidopsis* (Kohler et al., 1999; Kohler and Neuhaus, 2000) and tobacco (Arazi et al., 1999). Genome-wide identification of *CNGC* gene family was also conducted in *Arabidopsis* (Maser et al., 2001), rice (Bridges et al., 2005), *Populus trichocarpa* (Ward et al., 2009), pear (Chen et al., 2015), a moss (*Physcomitrella patens*) and some algae (Verret et al., 2010; Zelman et al., 2013). The *Arabidopsis CNGC* gene family is comprised of 20 members that are divided into groups I, II, III, IVa, and IVb according to their phylogenetic relationship (Maser et al., 2001). Recently, a plant CNGC-specific motif spanning the phosphate binding cassette (PBC) and hinge region within CNB domain of CNGC proteins is predicted. This motif identifies CNGCs but no other protein and thus represents an efficient tool to identify plant CNGCs, which has been validated in several species such as *Arabidopsis*, rice, *P. patens* and *Selaginella moellendorffii* (Zelman et al., 2012, 2013). Based on animal research, it has been suggested that plant CNGCs might conduct calcium. They might be activated by direct binding of cyclic nucleotides such as cAMP and cGMP to the CNB domain and inhibited by binding of CaM to the CaMB domain (Chin et al., 2009; Wang et al., 2013). It has been also suggested that upon activation, they promote influx of the calcium into the cytosol (Kaplan et al., 2007; Ma and Berkowitz, 2011). As a matter of fact, recent electrophysiological studies provide evidences that *AtCNGC18* functions as a Ca^2+^-permeable divalent cation-selective channel and is activated by cGMP and cAMP in HEK293T cells (Gao et al., 2014). Additionally, *AtCNGC18* is activated by a calcium-dependent protein kinase AtCPK32 in *Xenopus laevis* oocytes (Zhou et al., 2014).

The plant CNGCs are involved in numerous biological functions varying from plant development and stress tolerance (Kaplan et al., 2007) to disease resistance (Abdel-Hamid et al., 2011; Ma, 2011). As regulators of plant development and stress tolerance, *AtCNGC1* is involved in Ca^2+^ uptake (Ma et al., 2006); a *P. patens CNGC* (*PpCNGCb*) and its *Arabidopsis* ortholog (*AtCNGC2*) are essential to thermotolerance (Finka et al., 2012); *AtCNGC3* contributes to seed germination (Gobert et al., 2006); *AtCNGC6* mediates heat induced Ca^2+^ influx and the acquisition of thermotolerance (Gao et al., 2012); *AtCNGC10* is associated with plant growth (Borsics et al., 2007); *AtCNGC16* and *AtCNGC18* play a role in pollen fertility under stress and pollen tip growth respectively (Frietsch et al., 2007; Tunc-Ozdemir et al., 2013); while *AtCNGC19* and *AtCNGC20* are related to salt tolerance (Kugler et al., 2009). Additionally, genetics studies have unveiled the important role of four *Arabidopsis CNGC* genes, *AtCNGC2*, *AtCNGC4*, *AtCNGC11,* and *AtCNGC12*, in plant disease resistance. The *AtCNGC2* mutant, *defense*, *no death 1* (*dnd1*) exhibits diminished hypersensitive response (HR) development but shows enhanced basal resistance to *Pectobacterium carotovorum* (Ahn, 2007) as well as *R* gene-conferred resistance to avirulent pathogens with accumulation of salicylic acid (SA; Yu et al., 1998; Clough et al., 2000). *AtCNGC2* promotes Ca^2+^ current to produce nitric oxide (NO) which leads to HR generation in response to pathogen (Ali et al., 2007; Ma and Berkowitz, 2011). The *AtCNGC4* mutant, *defense*, *no death 2* (*DND2*)/*hypersensitive response-like lesion mimic 1* (*HLM1*) depicts a lesion mimic phenotype, shows constitutive pathogenesis-related (*PR*) gene expression and accumulates high level of SA (Balague et al., 2003; Jurkowski et al., 2004). This mutant displays resistance to *Pseudomonas syringae* pv. *tomato* (Pst) DC3000 but fails to induce HR after infection with avirulent strains of Pst and *Xanthomonas campestris* pv. *campestris* (Balague et al., 2003; Jurkowski et al., 2004). Mutation of *NEC1*, an *AtCNGC4* homolog in barley, leads to similar phenotypes to *dnd2/hlm1* (Rostoks et al., 2006; Keisa et al., 2011). *AtCNGC2* and *AtCNGC4* seem to work in the same signaling pathway in regulation of pathogen defense and floral transition (Chin et al., 2013). Additionally, the *Arabidopsis* mutant *constitutive expresser of pathogenesis related genes 22* (*cpr22*) in which a chimeric gene of *AtCNGC11* and *AtCNGC12* is generated, exhibits enhanced resistance to *Hyaloperonospora arabidopsidis* (formerly *Peronospora parasitica*; Yoshioka et al., 2001, 2006). The described evidence undoubtedly supports an essential role of CNGCs in plant defense. However, the evidence is mainly obtained from *Arabidopsis*. Studies in other plant species should promote understanding of CNGC functions.

In this study, we conducted genome-wide identification of CNGCs in the economically important crop tomato (*Solanum lycopersicum* L.) and explored function of the group IVb *SlCNGC* genes in various types of disease resistance. We demonstrated that tomato CNGC family is comprised of 18 members and group IVb *SlCNGCs* are required for a wide range of disease resistance in tomato.

## Materials and Methods

### Identification of *SlCNGC* Genes

The 20 *Arabidopsis* CNGC protein sequences were obtained from TAIR^1^ and were then used to TBLASTN search the tomato genome database^2^. All retrieved non-redundant sequences were collected and subjected to domain analysis using the programs Pfam^3^, SMART^4^ and CDD^5^. Those containing both a cyclic nucleotide-binding domain [CNBD, cNMP_binding family (PF00027)] and a TM or ion transport protein domain [ITP, Ion_trans family (PF00520)] were recognized as CNGC proteins. The PBC and hinge region within the CNB domain of all SlCNGCs were analyzed to check whether they carried a similar motif as suggested for plant CNGCs (Zelman et al., 2013).

### Cloning of *SlCNGC5*, *SlCNGC6,* and *SlCNGC15*

Leaves of 2-months-old tomato plants were collected from cultivars Heinz, Money maker and Suhong 2003. Total RNA extraction and reverse transcription were performed as before (Li et al., 2012). The obtained cDNA was used to amplify the *SlCNGCs*. As we expect that *SlCNGC6* is the combination of Solyc03g007260.2.1 and Solyc03g007250.2.1, which are annotated to be truncated N-part and C-part of a complete CNGC, we designed F and R primers corresponding to the beginning of Solyc03g007260.2.1 and the end of Solyc03g007250.2.1 respectively. For amplification of genes *SlCNGC5* and *SlCNGC15*, we designed F and R primers corresponding to the beginning and the end of Solyc06g051920.2.1 and Solyc03g098210.2.1 respectively. The primers were listed in Supplementary Table S1. PCR products were ligated into T-vector and sequenced. Sequences of the cloned SlCNGCs were compared with the corresponding loci in tomato genome database using GeneDoc program (Nicholas et al., 1997). The cloned SlCNGC sequences have been deposited at GenBank with accession numbers KJ499456, KJ499457, and KJ499458 for *SlCNGC5*, *SlCNGC6,* and *SlCNGC15* respectively.

### Gene Structure and Phylogenetic Analyses of *CNGCs*

Tomato and *Arabidopsis* CNGC protein sequences were aligned using clustalX 2.01 program (Larkin et al., 2007). The phylogenetic tree between these CNGCs was constructed using MEGA 5.0 (Tamura et al., 2011) with maximum likelihood (ML) method and bootstrap of 1000. The exon/intron structure of *CNGC* genes was analyzed online using the gene structure display server (GSDS)^6^.

### *Cis*-Acting Element Prediction of the *SlCNGC* Genes

*Cis*-acting element of 1 kb upstream sequence of the *SlCNGC* genes was predicted with Signal Scan search program in the PLACE database^7^.

### Gene Expression Analyses by qRT-PCR

Quantitative real time RT-PCR (qRT-PCR) analyses and consequent statistical data analyses were conducted as described (Zhao et al., 2013). The primers used in qRT-PCR analyses were listed (Supplementary Table S1). The primers for amplification of the *SlCNGC* genes were designed from their 5′ and 3′ UTR regions to ensure the gene targeting specificity.

### Plant Treatments and Inoculations

Tomato (cv. Money maker) plants were grown in growth room at 28°C with 16 h light/8 h dark photoperiod. For *SlCNGC* gene expression analyses, leaves of 2-months-old plants were sprayed with diverse chemicals including 380 μM benzothiadiazole (BTH), 10 mM ethephon (ETH), 200 μM jasmonic acid (JA), 375 μM oxalic acid (OA) or sterilized water as control.

For both *SlCNGC* gene expression and disease resistance evaluation analyses, tomato plants were inoculated with a variety of pathogens. *Sclerotinia sclerotiorum* was grown at 22°C on potato dextrose agar (PDA) medium for 2 days. The PDA plugs of 3 mm at diameter were taken from the colony outside circle that contained most active young mycelia, and then were stuck mycelial side down onto the tomato leaves. The inoculated plants were grown under high relative humidity for 24 and 4 h for disease resistance and expression analyses respectively. Inoculation with another fungal pathogen *Pythium aphanidermatum* and the bacterial pathogens *Pseudomonas syringae* pv. *tomato* (*Pst*) DC3000 and *Xanthomonas oryzae* pv. *oryzae* (*Xoo*) was performed as described (Li et al., 2012; Zhao et al., 2013).

For *SlCNGC* gene expression analyses, all the chemical-treated and pathogen-inoculated leaves were sampled at 4 h post treatment/inoculation and stored at -80°C.

### Pharmacological Studies

The chemicals (Sigma, USA) dibromo-cGMP (db-cGMP), dibutyryl-cAMP (db-cAMP), sodium metavanadate anhydrous (NaVO_3_), alloxan monohydrate and lanthanum (III) chloride hydrate (LaCl_3_) were dissolved into sterilized ddH_2_O while ethyleneglycol-bis-(2-aminoethyl ether)-*N*,*N*,*N*′,*N*′-tetraacetic acid (EGTA) into 1 M NaOH as stock solution. They were diluted with sterilized ddH_2_O in turn to 1 mM, 100 μM, 50 μM, 1 mM, 1 mM and 1 mM respectively, and infiltrated into tomato leaves with sterilized needless syringe. The infiltrated areas were immediately inoculated with *Ss* as mentioned above. The disease symptoms of the inoculated plants were investigated. Diameter of the disease lesions was measured by a ruler. In case of irregular lesions, the shortest and longest diameter of the same lesions was measured respectively and the average of them serves as the diameter of the lesions for statistical analysis.

### Virus-Induced Gene Silencing (VIGS) Analyses

The *SlCNGC* gene members are highly conserved among each other. Therefore care was taken to ensure the specificity to target an individual gene member. To achieve this, the VIGS target fragment of *SlCNGC16* and *SlCNGC17* were designed from 299 and 312 bp of 5′ UTR region while that of *SlCNGC18* corresponded to 144 bp of coding sequence. The cloning sites *Eco*RI and *Bam* HI were added to the end of the F and R primers for target fragment amplification respectively (Supplementary Table S1). These fragments were cloned and ligated into the TRV VIGS vector pYL156, which were subsequently electroporated into *Agrobacterium tumefaciens* strain GV3101 for VIGS analyses. VIGS analyses were conducted with vacuum-infiltration delivery approach as described (Wang et al., 2006; Cai et al., 2007) except that recombinant pYL156 with insertion of an eGFP fragment instead of an empty pYL156 was used as control to alleviate viral symptom (Cheng et al., 2012). At about 3 weeks post agro-infiltration, plants were inoculated with different pathogens and disease was investigated as described above. For each pathogen at least six silenced plants were examined. The experiments were conducted three times independently.

### Detection of PAMP/DAMP-Triggered H_2_O_2_ in Tomato Leaves

The peptides AtPep1 and flg22 with purity of over 95% were synthesized by China Peptides Company Limited. Tomato leaf disks of 3 mm at diameter were dipped in 200 μl of distilled water in a 96-well plate in dark over night. Water was replaced with 200 μl solution containing 100 μM luminal (Sigma-Aldrich) and 1 μg of horseradish peroxidase. AtPep1 (10 nM) and flg22 (100 nM) were added and immediately H_2_O_2_ were measured for 30 min as luminescence using a luminometer (Glomax 96 microplate luminometer, Promega).

## Results

### Treatment of cNMPs and Ca^2+^ Channel Related Chemicals Altered Resistance Against *Sclerotinia sclerotiorum* in Tomato

Pharmacological approach was employed to investigate the role of calcium channels in plant disease resistance. For this purpose a set of Ca^2+^ concentration regulators and putative Ca^2+^ channel activators and inhibitors including Ca^2+^ chelator EGTA; Ca^2+^ channel blocker LaCl_3_; Ca^2+^ ATPase blocker NaVO_3_; putative CNGC activators lipophilic dibromo-cGMP and dibutyryl-cAMP and adenylyl cyclase inhibitor alloxan (Ali et al., 2007; Ma et al., 2009; Qi et al., 2010), were infiltrated into tomato leaves, and subsequently effect of these chemicals on resistance to the important necrotrophic pathogen *S. sclerotiorum* was evaluated. Compared with controls, the necrosis disease symptom of tomato leaves infiltrated with 1 mM dibromo-cGMP, 100 μM dibutyryl-cAMP (Ma et al., 2009; Qi et al., 2010), and 50 μM NaVO_3_ was obviously less severe (Supplementary Figure S1A), indicated as significantly smaller diameter of lesions, 0.9 and 1.0 cm in cGMP- and cAMP-treated leaves while near 1.3 cm in non-treated control leaves respectively at 44 hpi (Supplementary Figure S1B). These results suggest that Ca^2+^ channels, which are regulated by cNMPs such as CNGCs, may play a role in tomato resistance to *S. sclerotiorum*.

This finding and the current status that function of CNGC has not been systemically studied in crop plant species raise our interest to identify at genome-wide level the CNGC family in economically important crop tomato and to dissect their roles in disease resistance.

### Identification of *CNGC* Genes in Tomato Genome

The 20 *Arabidopsis* CNGC protein sequences collected from TAIR^8^ were used to search against tomato genome with TBLASTN at Sol Genomics Network^9^. Consequently total 22 *SlCNGC* candidate genes were retrieved in tomato genome, which were subsequently analyzed for domain and motif composition. Those bear both a cyclic nucleotide-binding domain (CNBD) and a TM domain or an ion transporter (ITP) domain as well as a recently suggested plant CNGC specific motif (Zelman et al., 2013) are recognized as CNGC proteins. Domain composition analyses using three different databases including^10^, SMART^11^ and CDD^12^ revealed that out of the 22 sequences, five lacked one of the required domains. Among them, Solyc03g007250.1.1 and Solyc03g007260.2.1 contained a CNB domain and an ITP domain respectively. Moreover, they are adjacent on chromosome 3 with a small insertion of 383 bp. We thus suspect that mistake exists in these sequences and/or their annotations and they should be actually a full-length CNGC protein, which was later confirmed by our PCR cloning and sequencing results that will be described below. Similarly, another two sequences Solyc06g010180.1.1 and Solyc06g010190.1.1 carried a CNB domain and an ITP domain respectively, and located on the same chromosome (No. 6) but with a much larger insertion of 4506 bp. CDD analysis demonstrated that this fragment contained a GAG-pre-integrase domain (gag_pre-integrs [pfam13976]), an integrase core domain (rve [pfam00665]) and a gag-polypeptide of LTR copia-type retrotransposon (UBN2_2 [pfam14227]) (Supplementary Figure S2), indicating that a retrotransposon-mediated sequence insertion might have resulted in loss of a functional CNGC protein. Additionally, searching of a recently suggested plant CNGC specific motif in the PBC and hinge region (Zelman et al., 2013) revealed that the whole PBC and hinge region were deleted in Solyc06g051920.2.1 while the PBC only was deleted in Solyc03g098210.2.1 (Supplementary Figure S3). However, cloning of these two genes from tomato cv. Heinz 1706 and subsequent sequencing analyses demonstrated that the actual genes contained the whole PBC and hinge region, and thus the sequences deposited in SGN database were incorrect (see below for detail). Therefore, these two sequences are indeed full length tomato CNGCs. Finally, 18 full-length and three truncated CNGCs were identified in tomato genome (**Table 1**, Supplementary Table S2), which is less than in *Arabidopsis* (20; Supplementary Table S3; Maser et al., 2001). We named the *SlCNGCs* in ascending order in accordance with group numbers on the basis of phylogenetic relationship (see below for detail) for easier and better understanding of their functions.

**Table 1:** The SlCNGC gene family identified in this study.

Group	Gene symbol	Gene locus	Chromosomal position	Protein size (aa)	Intron	Domain organization			

						**Pfam**		**SMART**	**CDD**
						**Significant**	**Insignificant**		
I	SlCNGC1	Solyc01g095770.2.1	ch01 78724016-78720407	706	8	ITP, CNBD	–	cNMP, 5TMD,	CAP_ED
	SlCNGC2	Solyc05g050380.2.1	ch05 59634104-59638697	607	7	ITP, CNBD	–	cNMP, 3TMD,	ITP, CAP_ED
	SlCNGC3	Solyc05g050350.1.1	ch05 59612050-59608116	715	7	ITP, CNBD	–	cNMP, 5TMD,	ITP, CAP_ED
	SlCNGC4	Solyc05g050360.2.1	ch05 59616314-59621717	719	7	ITP, CNBD	–	cNMP, 5TMD,	ITP, CAP_ED
	SlCNGC5	Corrected Solyc06g051920.2.1	Corrected ch06 32082202-32086782	708	8	ITP, CNBD	—	cNMP, 3TMD,	ITP, CAP_ED
	SlCNGC6	Corrected (Solyc03g007260.2.1 + Solyc03g007250.1.1)	Corrected ch03 1817349-1813353 + ch03 1812970-1812278	708	6	ITP, CNBD	–	cNMP, 5 TMD	ITP, CAP_ED
II	SlCNGC7	Solyc07g005590.2.1	ch07 481459-487527	735	6	ITP, CNBD	–	cNMP, 5TMD	ITP, CAP_ED
	SlCNGC8	Solyc12g010010.1.1	ch12 3144264-3150904	692	5	ITP, CNBD	IQ^∗^	cNMP, 5TMD	ITP, CAP_ED
	SlCNGC9	Solyc03g116850.2.1	ch03 60167174-60162079	690	6	ITP, CNBD	IQ^∗^	cNMP, 5TMD	ITP, CAP_ED
III	SlCNGC10	Solyc11g069580.1.1	ch11 51297223-51293074	707	6	ITP, CNBD	IQ^∗^	cNMP, 6 TMD	ITP, CAP_ED
	SlCNGC11	Solyc09g007840.2.1	ch09 1376961-1373797	712	6	ITP, CNBD	–	cNMP, 5TMD	ITP, CAP_ED
	SlCNGC12	Solyc07g006510.2.1	ch07 1308919-1317697	720	6	ITP, CNBD	IQ^∗^	cNMP, 6TMD	ITP, CAP_ED
	SlCNGC13	Solyc08g069140.2.1	ch08 55418170-55422079	726	6	ITP, CNBD	–	cNMP, 5TMD	ITP, CAP_ED
	SlCNGC14	Solyc03g114110.2.1	ch03 58195300-58189455	678	5	ITP, CNBD	–	cNMP, 5TMD	CAP_ED
IVa	SlCNGC15	Corrected Solyc03g098210.2.1	Corrected ch03 54020551-54006686	763	12	CNBD	–	cNMP, 6TMD	ITP, CAP_ED
IVb	SlCNGC16	Solyc02g088560.2.1	ch02 45190069-45186246	708	7	CNBD	IC^∗^, IQ^∗^	cNMP, 6TMD,	CAP_ED
	SlCNGC17	Solyc10g006800.2.1	ch10 1246964-1240517	628	8	CNBD	GP41^∗^	cNMP, 6TMD	CAP_ED
	SlCNGC18	Solyc12g005400.1.1	ch12 242060-236128	686	7	CNBD	DUF4056^∗^	cNMP, 6TMD	CAP_ED, Neocle^∗^

*ITP, ion transport protein; CNBD, cyclic nucleotide-binding domain; IQ, IQ calmodulin-biding motif; IC, ion channel; GP41, glycoprotein 41; DUF, domain of unknown function; cNMP, cyclic nucleotide-monophosphate binding domain; TMD, transmembrane domain; CAP_ED, effector domain of CAP family. The asterisks showed in the table indicate the insignificant or incomplete domains*.

The SlCNGCs were basic proteins with a size of 607 amino acids (aa; SlCNGC2) ∼763 aa (SlCNGC15) that is dominated by about 700 aa (**Table 1**), which is similar to AtCNGCs (Supplementary Table S3; Maser et al., 2001).

### Domain and Motif Composition of *SlCNGC* Family

Domain composition analyses using databases Pfam, SMART and CDD revealed that in addition to the two main domains CNBD and TM or ITP, some tomato and *Arabidopsis* CNGC proteins contained several other insignificantly matched or incomplete domains and motifs (**Table 1**, Supplementary Table S3). These included IQ calmodulin-binding motif (PF00612) widely distributed in five SlCNGCs and seven AtCNGCs, a domain for GP41 family of envelop proteins from a variety of retroviruses (PF00517) in SlCNGC17 and AtCNGC2, TMEMspv1-c74-12 (PF11044) in a TM protein expressed by Plectrovirus spv1-c74 in AtCNGC2, and neocle (nucleoplasmin) domain (pfam03066) at N termini of SlCNGC18 and AtCNGC4, DUF4056 (PF13265) in SlCNGC18 and DUF4414 (pfam14377) in AtCNGC18. Whether these domains are actually functional awaits further experimental proof.

**FIGURE 1:**
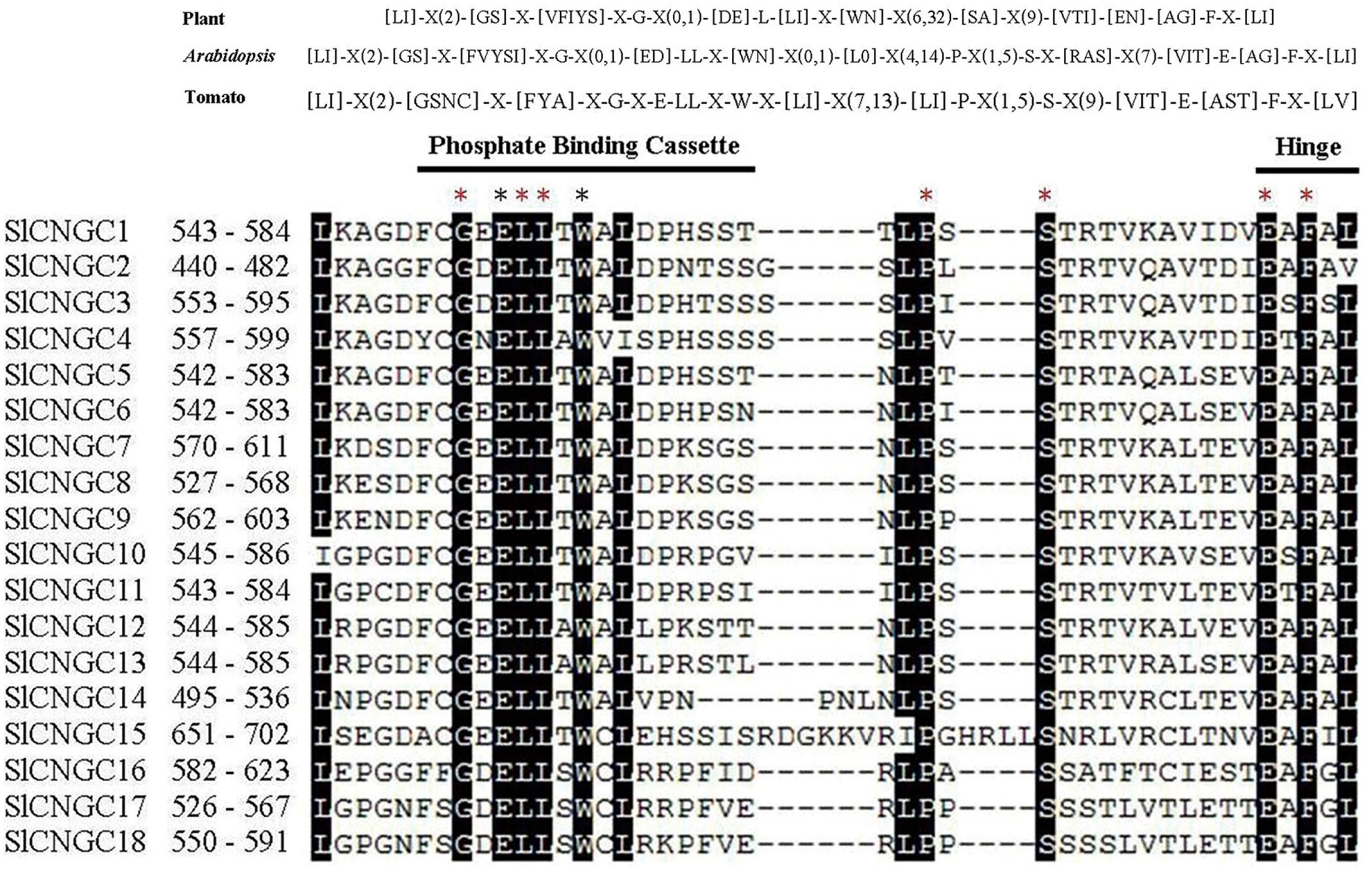
The cyclic nucleotide gated channel (CNGC)-specific motif spanning the phosphate binding cassette (PBC) and hinge region within CNB domain of 18 SlCNGCs. The CNGC-specific motifs for all plant species (Zelman et al., 2013) or *Arabidopsis* and tomato only are shown at top of alignment respectively. The square brackets “[ ]” indicate the amino acids allowed in this position of motif, “X” represents any amino acid, while round brackets “( )” denote the number of amino acids. The names and residue positions from the N-termini of SlCNGCs are indicated to the left of the motif. The PBC and hinge region are indicated by black lines above sequences. Residues in white highlighted in black indicate >90% conservation among SlCNGCs. The red and black asterisks above the alignment indicate 100% conservation among all tomato and *Arabidopsis* CNGCs and among tomato CNGCs only respectively. The SlCNGC-specific motifs were aligned by ClustalW and generated by MEGA5 program.

We further analyzed the PBC and hinge region within the CNB domain to check whether SlCNGCs carried similar motif as suggested for plant CNGCs (Zelman et al., 2013). The alignment of the PBC and hinge region of 18 SlCNGCs revealed a conserved motif in SlCNGCs as [LI]-X(2)-[GSNC]-X-[FYA]-X-G-X-E-LL-X-W-X-[LI]-X(7,13)-[LI]-P-X(1,5)-S-X(9)-[VIT]-E-[AST]-F-X-[LV] (**Figure 1**). Comparison of this motif with the one suggested by Zelman et al. (2013) for plant CNGCs, [LI]-X(2)-[GS]-X-[VFIYS]-X-G-X(0,1)-[DE]-L-[LI]-X-[WN]-X(6,32)-[SA]-X(9)-[VTI]-[EN]-[AG]-F-X-[LI], demonstrated that except for minor variation in some positions such as the 4th, 49th and the last positions, the motif for SlCNGCs generally fitted and was more conserved than that for plant CNGCs. All five essential amino acids G, L, S, E, and F in earlier version (Zelman et al., 2012) or three G, L, and F in their revised version (Zelman et al., 2013) of motif for plant CNGCs, presented in SlCNGCs as well. Furthermore, another four amino acids E, L, W, and P, which were not conserved in plant CNGCs, identically existed in all SlCNGCs (**Figure 1**). Alignment of the PBC and hinge region of 20 AtCNGCs gave rise to a motif as [LI]-X(2)-[GS]-X-[FVYSI]-X-G-X(0,1)-[ED]-LL-X-[WN]-X(0,1)-[L0]-X(4,14)-P-X(1,5)-S-X-[RAS]-X(7)-[VIT]-E-[AG]-F-X-[LI] with identical existence of seven amino acids G, L, L, P, S, E, and F (Supplementary Figure S4). Comparison of the PBC and hinge region of tomato and *Arabidopsis* CNGCs demonstrated that tomato CNGCs contained E and W two more identically existed amino acids in PBC (**Figure 1**, Supplementary Figures S4 and S5). These data reveal that the 18 SlCNGCs are most probably true CNGCs, and the PBC of CNB domain is more conserved within SlCNGCs than within AtCNGCs. Additionally, based on alignment of 38 tomato and *Arabidopsis* CNGCs, we predicted a more specific plant CNGC motif for the PBC and hinge region than the one suggested previously: [LI]-X(2)-[GSNC]-X-[FYVSIA]-X-G-X(0,1)-[ED]-LL-X-[WN]-X(0,1)-[LI0]-X(4,14)-P-X(1,5)-S-X(9)-[VIT]-E-[AGST]-F-X-[LIV] (Supplementary Figure S5). Whether this motif fits CNGCs of other plant species remains further analyses.

### Correction and Re-Annotation of Four Loci Deposited in SGN Database

As mentioned above Solyc03g007260.2.1 and Solyc03g007250.1.1 are localized on the same chromosome (No. 3) with a small insertion of 383 bp, which makes us hypothesize that they are actually two parts of the same *CNGC* gene. To confirm it, we designed forward and reverse primers separately starting from the start codon of Solyc03g007260.2.1 and the stop codon of Solyc03g007250.1.1 respectively, and finally the full-length sequence containing both parts was amplified by RT-PCR from tomato cv. Heinz 1706. Sequencing result revealed that the cloned gene carried an ORF of 2127 bp, rather not the total length of the insertion and Solyc03g007260.2.1 and Solyc03g007250.1.1 (2294 bp). It encoded for a protein of 708 aa (GenBank accession no. KJ499457, Supplementary Figure S6). Domain composition analysis showed that this protein possessed both CNB and IPT domains, and thus is a CNGC protein (**Table 1**). In accordance with the phylogenetic relationship with *Arabidopsis CNGCs*, we name this gene member as *SlCNGC6*. To check the possible variation of this gene in different tomato cultivars, we used the same primers to amplify this gene from the other two tomato cultivars Money maker and Suhong 2003, and obtained identical products to that from Heinz 1706 except one bp non-synonymous mutation (G^1642^ to C^1642^ resulting in C^548^ to S^548^) in Suhong 2003 (data not shown). Our data clearly show that *SlCNGC6* exists generally in tomato cultivars, and sequences and annotation of Solyc03g007260.2.1 and Solyc03g007250.1.1 deposited in SGN are incorrect.

Additionally, sequence analysis demonstrated that the whole PBC and hinge region or PBC only was deleted in Solyc06g051920.2.1 and Solyc03g098210.2.1 respectively (Supplementary Figure S3). To ensure the sequences, we cloned both full-length genes from Heinz 1706 with forward and reverse primers separately designed to start from the start codon and stop at the stop codon of Solyc06g051920.2.1 and Solyc03g098210.2.1 respectively. Consequently, we cloned ORFs of 2127 and 2292 bp. Sequencing results demonstrated that these cloned sequences were identical to Solyc06g051920.2.1 and Solyc03g098210.2.1 except that they were 207 and 111 bp longer respectively (GenBank accession no. KJ499456 and KJ499458, Supplementary Figures S7 and S8). Sequence comparison results revealed that our cloned sequences included the whole PBC and hinge regions (**Figure 1**, Supplementary Figure S5). We name these two genes as *SlCNGC5* and *SlCNGC15* respectively according to the phylogenetic relationship with *Arabidopsis CNGCs*. Our experimental data unanimously show that sequences of Solyc06g051920.2.1 and Solyc03g098210.2.1 deposited in SGN are not correct.

### Gene Structure and Chromosomal Location of *SlCNGC* Gene Family

To investigate further relationship between *SlCNGC* and *AtCNGC* genes, we comparatively analyzed their exon/intron structure (Supplementary Figure S9, Supplementary Table S4). In both *SlCNGC* and *AtCNGC* genes, the exon/intron structure predicted by GSDS exhibited group-specific feature in aspects of intron number and phase. Groups I, II, III, and IVb showed similar features, carrying 4∼8 phase 2 or 0 introns except *SlCNGC5*, while group IVa, carrying over nine introns, some of them belonging to phase 1 type (Supplementary Figure S9). These data suggested that expression and/or function of group IVa CNGCs might be different from the other CNGCs. When the exon/intron structure of the same group CNGC genes from tomato and *Arabidopsis* was compared, they displayed significant difference in intron size, which of tomato CNGC genes was dramatically larger than that of *Arabidopsis* counterparts. In addition, slight difference in the number and/or phase of introns of CNGC genes from the two species also existed (Supplementary Figure S9).

The 18 *SlCNGC* genes were distributed on 11 out of totally 12 chromosomes except chromosome 4 of tomato genome (**Table 1**). Four chromosomes bore two or more *SlCNGC* genes. Chromosomes 3 and 5 carried four and three *SlCNGCs* respectively, while chromosomes 7 and 12 carried two *SlCNGC* genes respectively. The other seven *SlCNGC* genes were solely located on seven different chromosomes respectively. Therefore, generally speaking, *SlCNGC* genes are evenly distributed in the whole tomato genome.

### Phylogenetic Relationship between Tomato and *Arabidopsis* CNGCs

**FIGURE 2:**
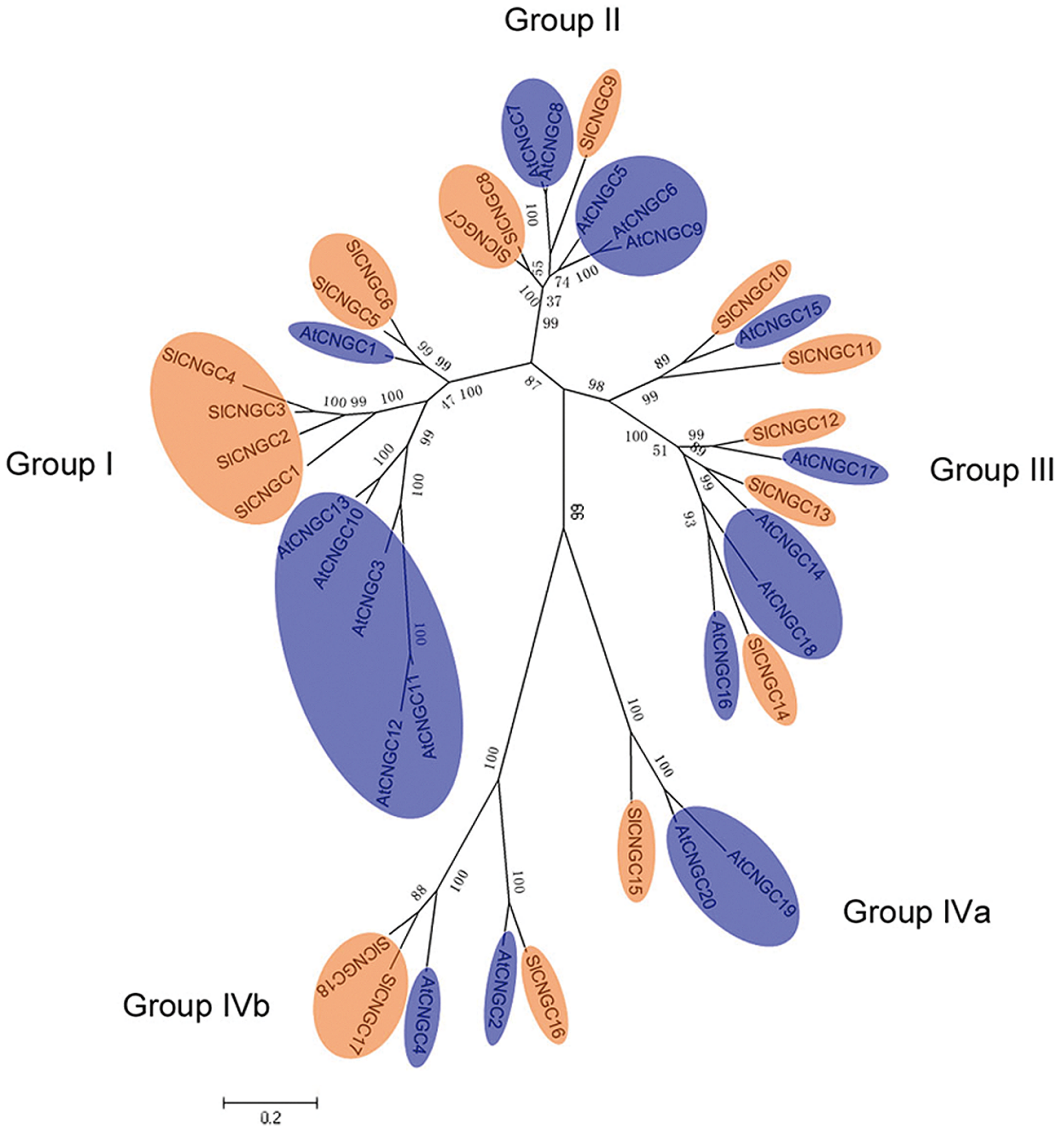
Phylogentic tree of the SlCNGC and AtCNGC proteins. The tree was created using Clustalx program by maximum liklihood (ML) method with bootstrap of 1000 in MEGA5. The SlCNGCs and AtCNGCs were shown in orange and blue respectively.

The relationship among *SlCNGC* proteins was observed by generating the unrooted ML phylogenetic tree. The 18 SlCNGCs were clustered in the tree into five groups (Supplementary Figure S10) as reported for AtCNGCs (Maser et al., 2001). To highlight the relationship between AtCNGCs and SlCNGCs, the ML phylogenetic tree was generated among 20 AtCNGCs (Maser et al., 2001) and 18 SlCNGCs. The SlCNGCs gathered with AtCNGCs into five groups (**Figure 2**). For each group of AtCNGCs tomato homologs existed. The size of each SlCNGC groups was unequal. The largest groups, groups I and III contained six and five members respectively; while the smallest one group IVa had just one member (**Figure 2**, Supplementary Figure S10). Compared with the group architecture of *Arabidopsis* CNGCs, group IVb of SlCNGCs gained one member; while groups II and IVa of SlCNGCs lost two and one member(s) respectively, which resulted in two member reduction in total in tomato CNGC family. In groups I and II, the tomato and *Arabidopsis* members clustered separately, implying that *CNGC* genes might have evolved separately in these two species.

### *Cis-*acting Element Composition of the *SlCNGC* Genes

In order to obtain preliminary clues for *SlCNGC* gene function, *cis*-acting elements in 1 kb upstream region of the *SlCNGC* genes were predicted in PLACE database. This divulged that *SlCNGC* promoter sequences generally carried a variety of predicted *cis*-acting elements that are responsive to various hormones such as ABA, auxin, GA, ethylene and cytokinin, biotic and abiotic factors and numerous WRKY transcription factors. However, the pattern of *cis*-acting elements in promoters of the different *SlCNGC* genes was distinct. Some elements widely distributed in promoters of almost all *SlCNGC* genes. Among them were nine elements responsive to ABA, auxin, GA, ethylene, cytokinin, biotic and abiotic factors respectively and four WRKY binding sites (**Table 2**). On the contrary, some elements were uniquely present in one *SlCNGC* gene. These included seven ABA responsive, two auxin responsive, one GA responsive and two biotic factor responsive elements. In addition, a Ca^2+^/CaMB element CGCGBOXAT existed in the promoters of *SlCNGC2* and *SlCNGC11*, suggesting that these two *SlCNGC* genes might be feedback regulated by Ca^2+^/calmodulin. Interestingly, promoters of *SlCNGC17* and *SlCNGC18*, two group IVb *SlCNGC* genes, contained biotic factor-regulated elements, indicating that these *SlCNGC* genes might play important roles in regulating plant disease resistance. Collectively, the element-profiling analysis revealed that *SlCNGC* genes might be regulated widely by a variety of factors including phytohormones, biotic and abiotic factors, and the underlying regulation mechanisms vary for different *SlCNGC* members.

**Table 2:** Predicted *cis*-acting elements in 1 kb upstream region of the *SlCNGC* gene family.

Regulator	*Cis*-acting element	Signal sequence	Code	Number of elements in 18 *SlCNGCs*																	
				1	2	3	4	5	6	7	8	9	10	11	12	13	14	15	16	17	18
ABA	ABREATCONSENSUS	YACGTGGC	S000406	0	0	0	0	0	0	0	0	0	0	0	0	1	0	0	0	0	0
	ACGTABREMOTIFA2OSEM	ACGTGKC	S000394	0	0	0	0	0	0	0	0	0	0	0	0	1	0	0	0	0	0
	ABREATRD22	RYACGTGGYR	S000013	0	0	0	0	0	0	0	0	0	0	0	0	1	0	0	0	0	0
	ATHB6COREAT	CAATTATTA	S000399	0	1	0	0	0	0	0	0	0	0	0	0	0	0	0	0	0	0
	DRE2COREZMRAB17	ACCGAC	S000402	0	0	0	0	1	0	0	0	0	0	0	0	0	0	0	0	0	0
	DPBFCOREDCDC3	ACACNNG	S000292	1	0	2	2	0	0	0	0	0	0	0	0	0	0	0	2	0	0
	MYCATRD22	CACATG	S000174	2	1	1	0	0	1	1	0	0	0	0	1	1	1	0	2	0	0
	MYB2CONSENSUSAT	YAACKG	S000409	2	0	1	0	1	2	3	1	2	0	0	0	1	0	0	1	0	1
	MYBATRD22	CTAACCA	S000175	0	0	0	0	0	0	0	0	0	1	0	0	0	1	0	0	0	0
	PYRIMIDINEBOXHVEPB1	TTTTTTCC	S000298	0	0	0	2	1	0	0	0	1	0	0	0	0	0	2	0	0	0
	PROXBBNNAPA	CAAACACC	S000263	0	0	0	0	0	0	0	0	0	0	0	0	0	0	0	0	1	0
	RYREPEATBNNAPA	CATGCA	S000264	2	1	1	0	0	0	0	0	4	0	0	0	0	0	0	0	0	0
	RYREPEATVFLEB4	CATGCATG	S000102	0	0	0	0	0	0	0	0	2	0	0	0	0	0	0	0	0	0
Auxin	ARFAT	TGTCTC	S000270	0	0	1	0	3	2	0	0	0	0	0	0	0	0	0	0	0	2
	ASF1MOTIFCAMV	TGACG	S000024	1	0	0	0	0	2	0	1	2	0	1	1	0	0	0	2	0	0
	AUXREPSIAA4	KGTCCCAT	S000026	0	0	0	0	0	0	0	0	0	0	0	0	0	0	0	0	0	1
	GGTCCCATGMSAUR	GGTCCCAT	S000360	0	0	0	0	0	0	0	0	0	0	0	0	0	0	0	0	0	1
	NTBBF1ARROLB	ACTTTA	S000273	1	0	4	1	0	2	1	1	1	1	0	1	3	0	4	1	1	2
GA	GARE1OSREP1	TAACAGA	S000419	0	1	0	0	0	0	0	0	1	0	0	0	0	1	0	0	0	0
	GARE2OSREP1	TAACGTA	S000420	0	0	1	0	0	0	0	0	0	0	0	0	0	0	0	0	0	0
	GAREAT	TAACAAR	S000439	0	1	0	0	0	1	1	0	1	4	0	0	0	4	2	1	1	1
	MYBGAHV	TAACAAA	S000181	0	0	0	0	0	0	0	0	0	3	0	0	0	3	2	1	1	0
	PYRIMIDINEBOXOSRAMY1A	CCTTTT	S000259	3	1	0	3	2	1	0	1	1	0	0	0	0	0	2	1	2	1
	TATCCAOSAMY	TATCCA	S000403	1	0	3	2	0	2	3	0	0	0	1	0	1	0	0	0	2	0
	TATCCACHVAL21	TATCCAC	S000416	0	0	0	1	0	0	1	0	0	0	0	0	0	0	0	0	0	0
ETH	ERELEE4	AWTTCAAA	S000037	1	1	0	0	2	1	2	0	2	0	1	1	1	1	0	1	1	1
	LECPLEACS2	TAAAATAT	S000465	0	0	0	0	0	0	0	0	0	1	0	0	1	0	2	2	0	0
	ARR1AT	NGATT	S000454	9	11	11	10	14	15	21	10	21	12	13	8	9	13	5	11	7	12
Cytokinin	CPBCSPOR	TATTAG	S000491	0	0	1	0	1	3	0	1	0	0	1	2	1	1	1	0	2	2
Ca^2+^/Calmodulin binding	CGCGBOXAT	VCGCGB	S000501	0	2	0	0	0	0	0	0	0	0	2	0	0	0	0	0	0	0
Biotic	BOXLCOREDCPAL	ACCWWCC	S000492	0	0	0	0	0	1	0	0	0	1	0	0	0	0	1	0	0	0
	CACGTGMOTIF	CACGTG	S000042	0	0	0	0	0	0	0	0	0	0	0	0	0	1	0	0	0	0
	ELRECOREPCRP1	TTGACC	S000142	1	0	2	1	0	1	2	0	2	0	0	1	0	0	0	0	0	0
	GT1GMSCAM4	GAAAAA	S000453	2	2	1	7	2	2	1	0	1	3	3	5	7	1	6	3	7	3
	MYB1LEPR	GTTAGTT	S000443	0	2	0	0	0	0	0	0	0	1	0	1	0	0	0	0	0	0
	QARBNEXTA	AACGTGT	S000244	1	0	0	0	0	0	0	0	0	0	0	0	0	0	0	0	0	0
	SEBFCONSSTPR10A	YTGTCWC	S000391	1	0	0	0	4	3	0	0	0	0	0	0	0	0	0	0	0	0
Abiotic	MYCATERD1	CATGTG	S000413	2	1	1	0	0	1	1	0	1	1	0	1	1	1	0	2	0	0
	MYCCONSENSUSAT	CANNTG	S000407	10	6	41	8	4	10	6	8	6	4	10	6	6	10	2	10	0	0
	MYB2AT	TAACTG	S000177	0	0	0	1	1	1	0	2	0	1	0	0	0	0	0	0	0	0
WRKY transcription factor	WBBOXPCWRKY1	TTTGACY	S000310	1	1	3	2	1	0	0	0	0	1	0	1	0	2	0	0	0	0
	WBOXATNPR1	TTGAC	S000390	4	2	5	4	2	3	4	4	4	3	1	2	2	6	0	0	0	1
	WBOXHVISO1	TGACT	S000442	5	2	3	3	3	1	4	3	4	4	0	3	1	4	1	2	1	1
	WBOXNTERF3	TGACY	S000457	5	2	5	4	3	2	7	3	7	6	0	5	2	4	2	2	3	1
	WRKY71OS	TGAC	S000447	9	4	9	5	4	7	7	9	7	7	4	7	3	9	4	5	4	5
	WBOXNTCHN48	CTGACY	S000508	1	1	0	0	0	1	1	0	1	0	0	1	0	1	2	0	2	0

### Expression Pattern of the *SlCNGC* Genes in Response to Diverse Stimuli

**FIGURE 3:**
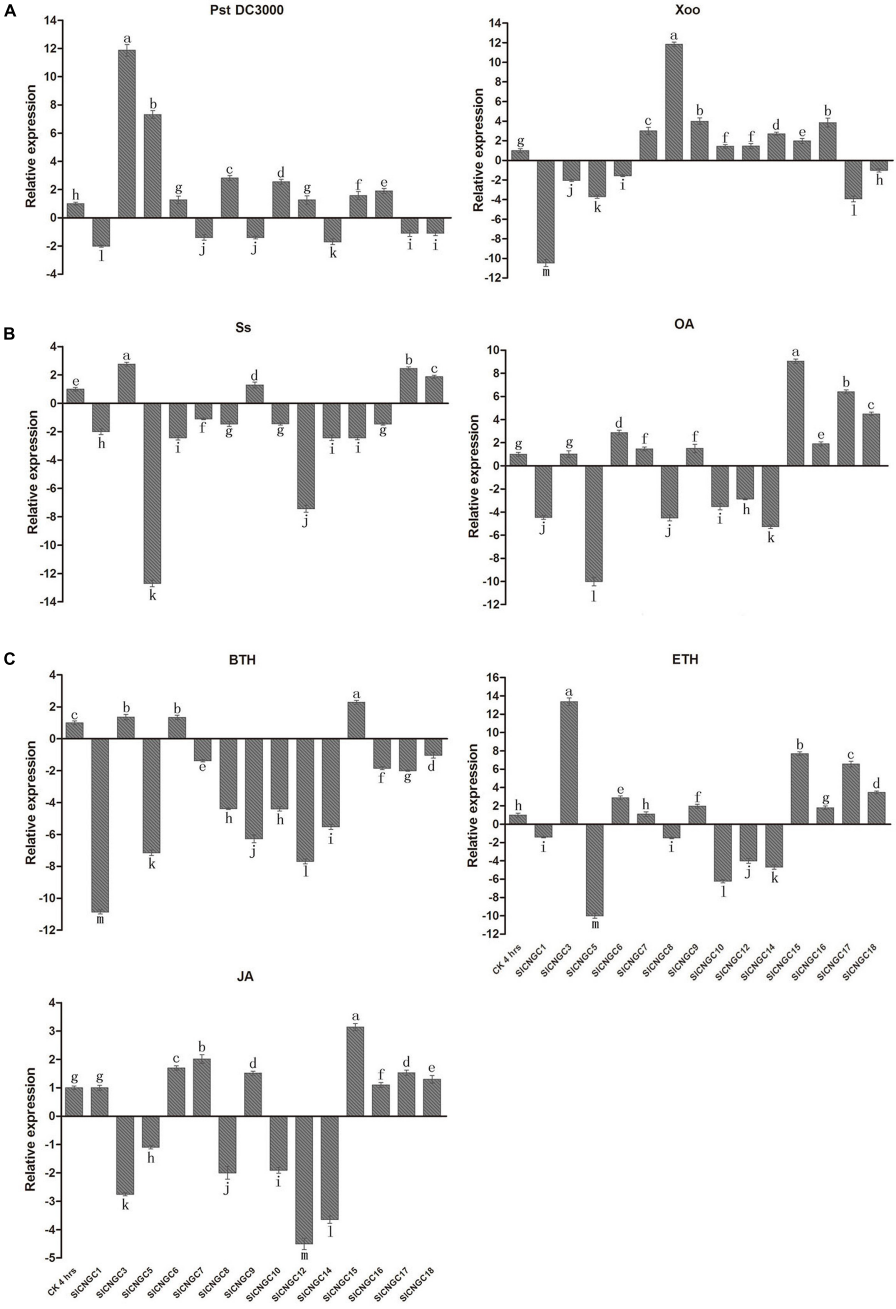
Expression profiles of 14 *SlCNGC* genes. Expression profiles of the *SlCNGC* genes in response to *Pst* DC3000 and *Xoo* inoculation **(A)**, oxalic acid (OA) treatment and *Ss* inoculation **(B)** and hormone treatments **(C)** were detected by qRT-PCR with the gene-specific primers listed in Supplementary Table S1. The small letters indicated the significant difference among expression level of the *SlCNGC* genes in treated samples and non-treated CK and data represent the mean ± SE of three independent experiments (*p* < 0.05, DMRT).

By quantitative real-time PCR (qRT-PCR) analysis, the expression patterns of *SlCNGC* genes were examined in early response (4 hpi) to various defense signaling molecules or their analogs or compounds from which they are released, including ethylene-releasing compound ETH, JA and a biologically active analog of SA BTH, the pathogen *S. sclerotiorum* (*Ss*)-secreted phytotoxin OA and the pathogens *Ss*, *Pst* DC3000 and *Xoo* in tomato (**Figure 3**). The specific primers used for qRT-PCR were listed in Supplementary Table S1. The gene expression patterns were distinct among different *SlCNGC* genes in response to diverse stimuli. When viewed from the stimuli, *Ss* inoculation and BTH treatment down-regulated expression of most of the *SlCNGC* genes, while the other stimuli up-regulated expression of eight *SlCNGC* genes but down-regulated expression of the other six *SlCNGC* genes. When analyzed from the *SlCNGC* genes, expression of group III *SlCNGC* genes was suppressed by BTH, ETH, JA, and OA treatments and *Ss* inoculation while was not significantly altered or induced by *Pst* DC3000 and *Xoo* inoculation except *SlCNGC14* by *Pst* DC3000 inoculation. For group IVb genes, *SlCNGC17* and *SlCNGC18* exhibited same expression patterns, both up-regulated by ETH, JA, and OA treatment and *Ss* inoculation while down-regulated by BTH treatment and *Pst* DC3000 and *Xoo* inoculation. The remaining member *SlCNGC16* displayed similar expression pattern except responsiveness to three pathogens, which was identical to *SlCNGC15*, the sole member of group IVa. For groups I and II, different members showed distinct expression patterns in response to the stimuli. *SlCNGC5* was generally highly responsive to almost all stimuli tested in this study. *SlCNGC3* specially and highly responded to *Pst* DC3000 inoculation and ETH treatment, while *SlCNGC1* and *SlCNGC8* highly responded to *Xoo* inoculation and OA and BTH treatments. The expression data indicated that some *SlCNGC* genes may play a role in regulating plant disease resistance.

### Silencing of Group IVb *SlCNGC* Genes Significantly Enhanced Resistance to Necrotrophic Pathogens but Reduced Resistance to Viral Pathogen TRV

**FIGURE 4:**
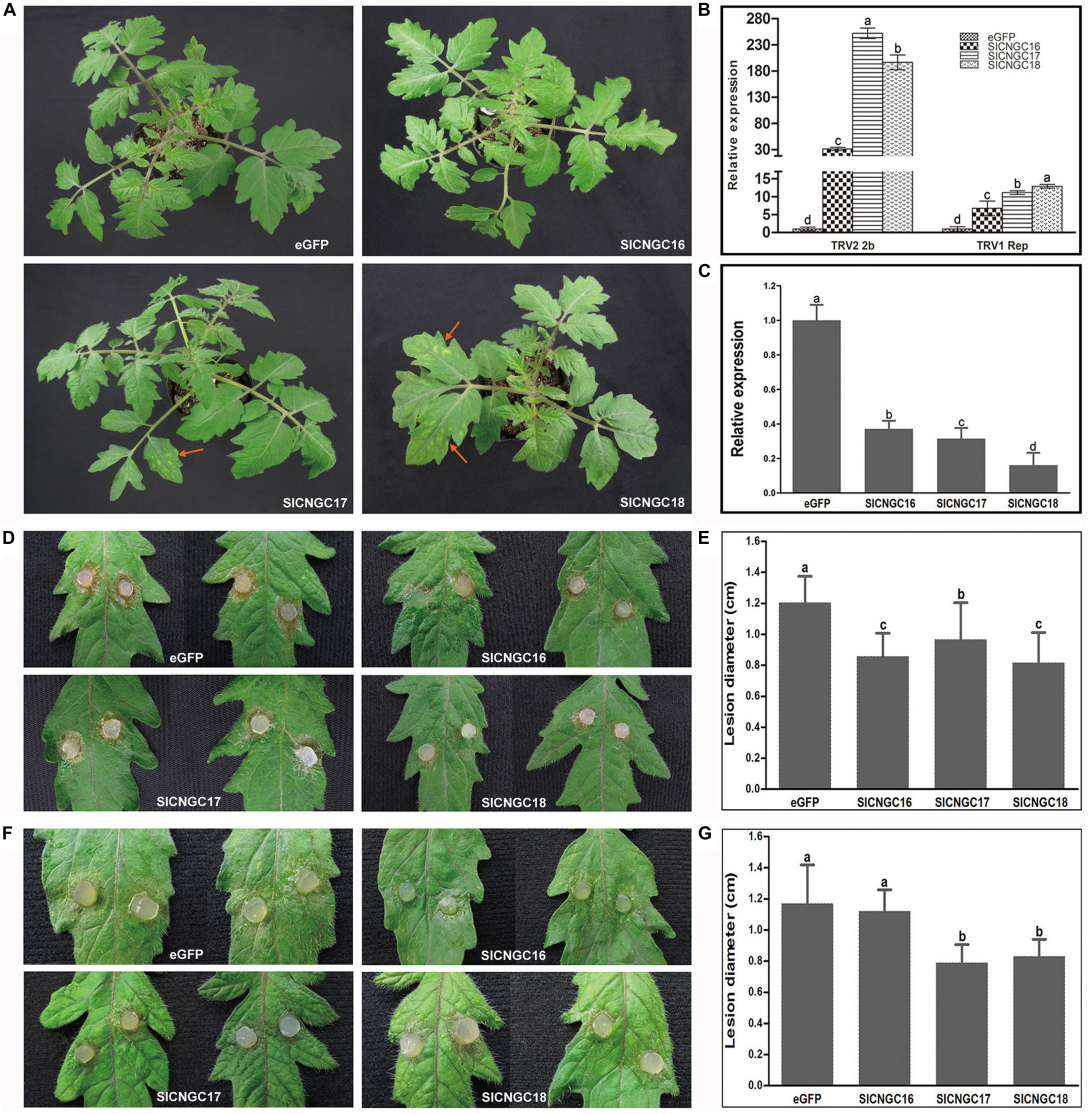
Silencing of group IVb *SlCNGC* genes in tomato plants reduced resistance to *Tobacco rattle virus* (TRV) and enhanced resistance to *Sclerotinia sclerotiorum* and *Pythium aphanidermatum*. **(A)** Phenotypes of the *SlCNGC*-silenced plants. Gene silencing analyses were performed using TRV-based vectors. TRV viral symptoms (indicated with red arrows) were more severe in the *SlCNGC17-* and *SlCNGC18-*silenced plants compared with the eGFP-control plants. Photographs were taken 3 weeks post agro-inoculation. **(B)** Detection of transcripts of the TRV_1_ replicase gene and TRV_2_ 2b gene in silenced plants by qRT-PCR. **(C)** Gene expression anaysis by qRT-PCR to detect *SlCNGC* gene silencing efficiency. **(D)** Disease symptoms of the *SlCNGC*-silenced plants after inoculated with *S. sclerotiorum* (*Ss*). Photograhps were taken at 24 hpi. **(E)** Statistical anaysis of disease severity in *Ss* inoculated plants. **(F)** Disease symptoms of the *SlCNGC*-silenced plants after inoculated with *P. aphanidermatum*. Photograhps were taken at 14 hpi. **(G)** Statistical anaysis of disease severity in *P. aphanidermatum* inoculated plants. For each pathogen at least six silenced plants were examined. The experiments were conducted three times independently. The data in all statistical analyses represent the mean ± SD of three experiments. Significant difference is indicated as small letters (*p* < 0.05, DMRT).

Further detailed functional analyses of group IVb *SlCNGC* genes in plant disease resistance were performed employing virus-induced gene silencing (VIGS) technique. To ensure the target specificity of silencing, the gene-specific UTR sequences of the *SlCNGC* genes were used for VIGS analyses. They were ligated into the TRV silencing vector pYL156 for silencing analyses, while non-silenced eGFP fragment-inserted recombinant pYL156 vector was used as a negative control (Cheng et al., 2012). Three weeks after agro-infiltration for silencing, the tomato plants agro-infiltrated for pYL156::SlCNGC17 and pYL156::SlCNGC18 showed clear mosaic symptoms in leaves; those for pYL156::SlCNGC16 showed very mild mosaic symptoms, while those for control vector pYL156::eGFP grew normally **Figure 4A**). Gene expression analysis revealed that transcripts of TRV_1_ replicase gene accumulated 6, 11, and 12 folds higher in *SlCNGC16*-, *SlCNGC17*- and *SlCNGC18*-silenced plants respectively than control plants. More strikingly, transcripts of TRV_2_ 2b gene accumulated 31, 252, and 196 folds higher in *SlCNGC16*-, *SlCNGC17*-, and *SlCNGC18*-silenced plants respectively than control plants (**Figure 4B**).

To clarify whether the *SlCNGC* genes had been efficiently silenced in the agro-infiltrated plants, transcripts of these genes in the agro-infiltrated plants were quantified with qRT-PCR. Result showed that transcripts of *SlCNGC16*, *SlCNGC17,* and *SlCNGC18* in the agro-infiltrated plants dropped to 37, 31, and 17%, respectively, of that in control plants (**Figure 4C**). These results demonstrated that silencing of these *SlCNGC* genes resulted in the TRV viral symptoms and higher level of virus accumulation. Together, these data indicated that the group IVb *SlCNGC* genes, especially *SlCNGC17* and *SlCNGC18* positively regulate tomato resistance to TRV.

To further probe functions of group IVb *SlCNGCs* in disease resistance, the silenced tomato plants were inoculated with a set of different types of pathogens. The non-host bacterial semi-biotrophic pathogen, *Xoo*, the host bacterial semi-biotrophic pathogen *Pst* DC3000 and the host fungal necrotrophic pathogens *Ss* and *P. aphanidermatum* were tested. Non-host resistance to *Xoo* in silenced tomato plants for all three *SlCNGC* genes was similar to that in control plants, as both plants developed clear HR necrosis in infiltrated areas at 14 hpi (Supplementary Figure S11A). Resistance to *Pst* DC3000 was also not altered significantly in silenced plants when compared with the control plants. Both plants showed severe necrosis at 48 hpi (Supplementary Figure S11B). However, when inoculated with *Ss*, necrotic symptoms of the leaves of the *SlCNGC*-silenced plants were obviously less severe than that of the eGFP-control plants (**Figure 4D**). The lesions in the *SlCNGC*-silenced plants, lower than 1.0 cm in diameter, were statistically significantly smaller than those in control plants, 1.2 cm in diameter at 24 hpi (**Figure 4E**).

When investigated with another important necrotrophic pathogen *P. aphanidermatum*, results similar to *Ss* were observed except *SlCNGC16*. After inoculation with *P. aphanidermatum*, pathogen-driven necrosis in the leaves of *SlCNGC17*- and *SlCNGC18*-silenced plants were obviously less severe than that of the eGFP-control plants (**Figure 4F**). The necrotic lesions in the silenced plants, about 0.8 cm in diameter, were strikingly smaller than those in control plants, near 1.2 cm in diameter at 14 hpi (**Figure 4G**). However, leaves of the *SlCNGC16*-silenced plants displayed necrotic lesions approximately with similar size to those of the eGFP-control plants (**Figures 4F,G**). These data revealed that the group IVb *SlCNGCs* negatively regulates resistance to necrotrophic pathogens *Ss* and *P. aphanidermatum* in tomato plants.

Collectively, our results reveal that the group IVb *SlCNGC* genes play important roles in regulation of plant disease resistance to a variety of pathogens in a gene- and target pathogen-dependent manner. *SlCNGC17* and *SlCNGC18* negatively regulate resistance to necrotrophic pathogens such as *Ss* and *P. aphanidermatum* while positively regulate resistance to viral pathogens such as TRV in tomato plants. However, *SlCNGC16* mainly plays a role in regulating resistance to the necrotrophic pathogen *Ss*.

### Silencing of Group IVb *SlCNGC* Genes Reduced PAMP- and DAMP-Triggered Hydrogen Peroxide Accumulation

**FIGURE 5:**
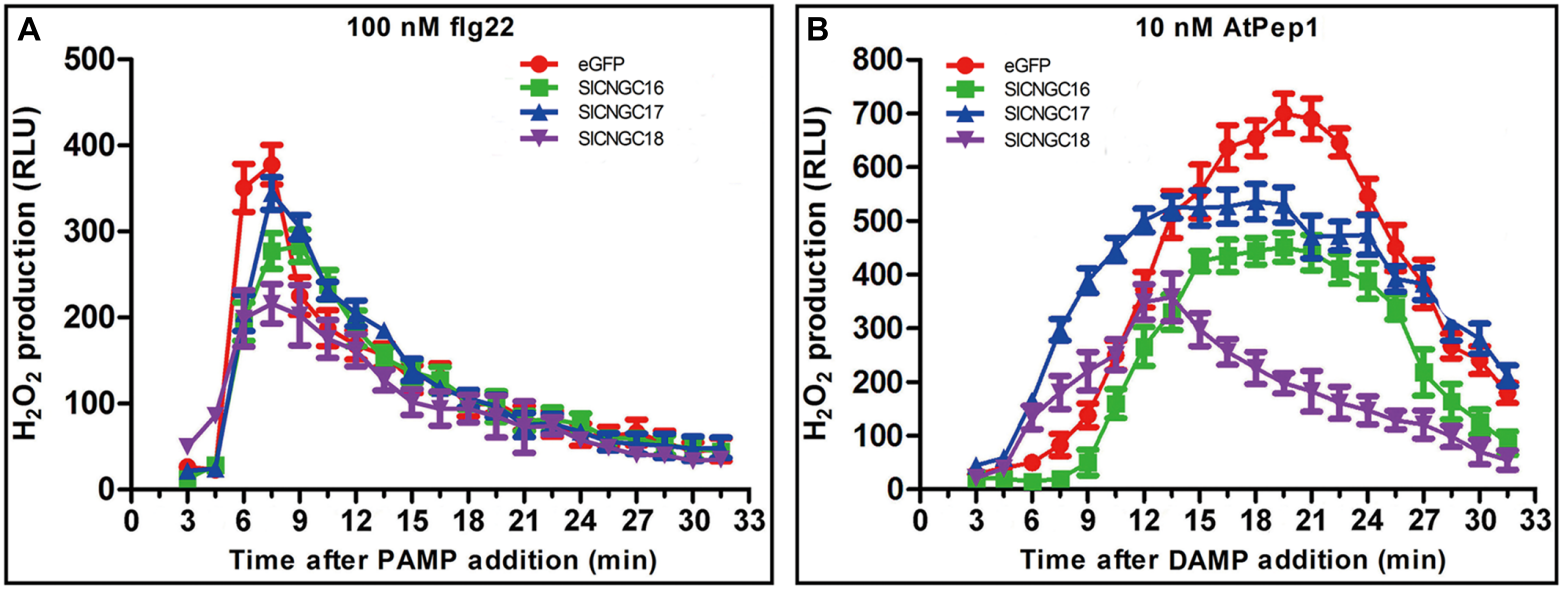
Silencing of group IVb *SlCNGC* genes in tomato plants reduced PAMP/DAMP-induced accumulation of H_**2**_O_**2**_. Production of H_2_O_2_ induced by 100 nM flg22 **(A)** and 10 nM AtPep1 **(B)** was detected using a luminal-based assay in leaf disks of the *SlCNGC16-*, *SlCNGC17-,* and *SlCNGC18*-silenced plants and the eGFP non-silenced control plants. Data are shown as relative luminal units (RLU) and represent the mean ± SE of three independent experiments.

To further explore functions of *SlCNGCs* in disease resistance, role of group IVb *SlCNGC* genes in pathogen-associated molecular pattern (PAMP)- and damage-associated molecular pattern (DAMP)-triggered immunity was examined. AtPep1 (10 nM) and flg22 (100 nM) were applied to leaf disks (3 mm at diameter) of the *SlCNGC*-silenced and non-silenced (control) plants. Subsequently the dynamics of hydrogen peroxide (H_2_O_2_) accumulation, which was indicated as relative luminescence (RLU), was measured. In response to flg22, H_2_O_2_ increased rapidly and peaked at 7 min post application (mpa) to 377 RLU and subsequently decreased quickly to reach the basal level within about 30 mpa in control plants (**Figure 5A**). In the *SlCNGC*-silenced plants, H_2_O_2_ accumulation displayed similar dynamics but the peak value was lower, which differed depending on the *SlCNGC* gene that was silenced. In the *SlCNGC16*- and *SlCNGC18*-silenced plants, H_2_O_2_ peaked to 277 and 216 RLU respectively, which was significantly lower than control. However, in the *SlCNGC17*-silenced plants, H_2_O_2_ peaked to 344 RLU, which was only slightly lower than control (**Figure 5A**). These data supported a positive role of group IVb *SlCNGC* genes, especially *SlCNGC16* and *SlCNGC18* in PAMP-triggered immunity (PTI). After application of AtPep1, H_2_O_2_ accumulation peaked to 700 RLU at 20 mpa in control plants, 451 RLU at 20 mpa in the *SlCNGC16*-silenced plants, 530 RLU at 20 mpa in the *SlCNGC17*-silenced plants while 349 RLU at 15 mpa in the *SlCNGC18*-silenced plants (**Figure 5B**), demonstrating that silencing of group IVb *SlCNGC* genes, especially *SlCNGC16* and *SlCNGC18*, strongly reduced the AtPep1-triggered H_2_O_2_ accumulation, and therefore suggesting that the group IVb *SlCNGC* genes are required for PTI amplification triggered by plant endogenous DAMPs.

### Silencing of Group IVb *SlCNGC* Genes Reduced Expression of the Defense-Related Ca^2+^ Signaling Genes

**FIGURE 6:**
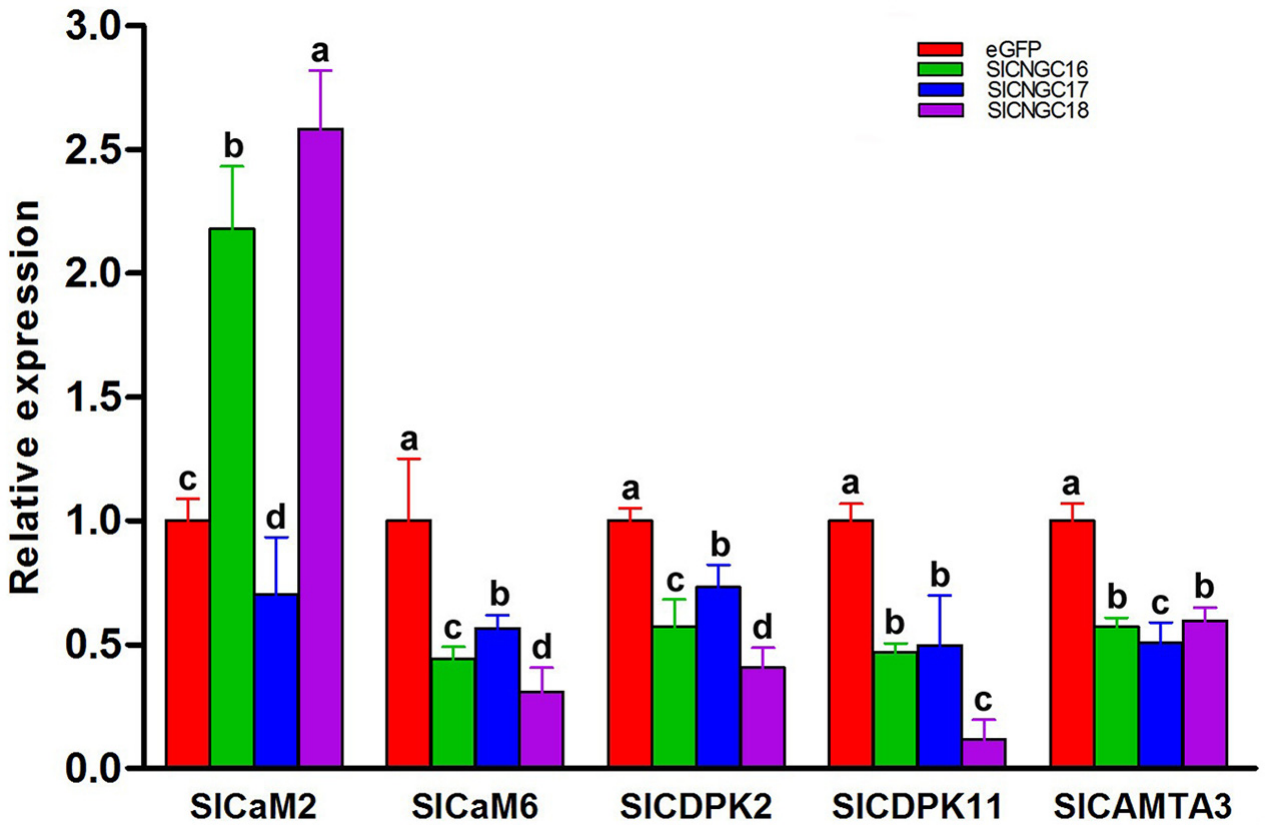
Expression of a set of Ca^**2+**^ signaling genes in group IVb *SlCNGC*-silenced tomato plants. Expression of *SlCaM2*, *SlaM6*, *SlCDPK2*, *SlCDPK11,* and *SlCAMTA3* genes in the *SlCNGC16-*, *SlCNGC17-,* and *SlCNGC18*-silenced plants and the eGFP non-silenced control plants was analyzed by qRT-PCR with gene-specific primers listed in Supplementary Table S1. Small letters denote the significant difference between expression values of the silenced plants and those of the eGFP-control plants (*p* < 0.05, DMRT). Data represent the mean ± SE of three independent experiments.

To find out molecular mechanism of group IVb *SlCNGC* genes in regulating plant disease resistance, we examined expression of a series of resistance-related Ca^2+^ signaling genes to clarify whether they are involved in group IVb *SlCNGCs*-mediated resistance regulation. The genes under this expression analysis included two calmodulin genes *SlCaM2* and *SlCaM6*, two calcium-dependent protein kinase genes S*lCDPK2* and *SlCDPK11*, which are the tomato homologs of *Arabidopsis AtCPK2* and *AtCPK11*, and one CaM-binding transcription activator gene *SlCAMTA3*. All these genes play an important role in regulating disease resistance (Du et al., 2009; Boudsocq et al., 2010; Zhao et al., 2013). The expression result depicted that silencing of all three group IVb *SlCNGC* genes *SlCNGC16*, *SlCNGC17,* and *SlCNGC18* unanimously reduced the expression of *SlCaM6*, *SlCDPK2*, *SlCDPK11,* and *SlCAMTA3*, but differently affected the expression of *SlCaM2*. Silencing of *SlCNGC16* and *SlCNGC18* increased its expression by 2.2 and 2.6 folds respectively, while silencing of *SlCNGC17* reduced its expression by 70% (**Figure 6**). These results indicated that CaMs, CDPKs, and CAMTAs may play a role in the group IVb *SlCNGCs*-mediated resistance.

## Discussion

### Tomato and *Arabidopsis* CNGC Families

Cyclic nucleotide gated channel is suggested to be Ca^2+^ conducting channel in plants, yet, surprisingly, genome-wide identification and functional study so far are mainly conducted in the model plants *Arabidopsis*. In this study, we have identified CNGC family in the economically important crop plant tomato. Through bioinformatics and experimental analyses, we finally identified 18 tomato CNGCs. It is unexpected that members of CNGCs in tomato are less than in *Arabidopsis*, which contains 20 members (Maser et al., 2001), considering that the genome size of tomato is much larger than *Arabidopsis*. It seems unlikely that our identification is incomplete, since only 22 sequences were retrieved when we performed BLAST searching with all 20 AtCNGCs. All 22 retrieved sequences had been analyzed in detail. More importantly, for those annotated to be truncated, confirmation experiments were conducted such as RT-PCR cloning and sequencing. Consequently, we have corrected four loci in SGN tomato database, including completion of Solyc06g051920.2.1 and Solyc03g098210.2.1 sequences and naming them as SlCNGC5 and SlCNGC15 respectively, and reannotating two truncated sequences Solyc03g007260.2.1 and Solyc03g007250.1.1 as a full-length CNGC (SlCNGC6; **Table 1**, Supplementary Figures S6–S8). Then what are the reasons to cause smaller CNGC family in tomato than in *Arabidopsis*? We find that a gag-polypeptide of LTR copia-type retrotransposon presents between two truncated sequences Solyc06g010180.1.1 and Solyc06g010190.1.1 (Supplementary Figure S2). Moreover, SlCNGC17 contains a domain homologous to GP41 family of retroviral envelop protein (**Table 1**). Therefore, it is possible that retrotransposons and retroviruses disrupt *CNGC* genes and lead to loss of full-length *CNGC* gene members in tomato genome. Additionally, smaller CNGC family in tomato than in *Arabidopsis* could be ascribed to relatively more conserved genomes among the members of *Solanaceae* family, including tomato (Mueller et al., 2005). Moreover, genome size of rice and pear is significantly larger than that of *Arabidopsis* but they are comprised of only 16 and 21 CNGC genes respectively (Nawaz et al., 2014; Chen et al., 2015). This indicates that the phenomenon that a species with a larger genome contains similar or even less CNGC genes in comparison with *Arabidopsis* also occurs in plant species other than tomato. The cause and possible effect of this phenomenon remains further dissection.

The phylogenetic and gene structure analyses manifest that generally tomato *CNGC* genes exhibit group-wise similar structure and clusters to *Arabidopsis* counterparts (Supplementary Table S4; **Figure 2**, Supplementary Figures S9 and S10). However, *CNGC* genes of the two species discriminate in the following aspects. Firstly, the group I and II AtCNGCs and SlCNGCs cluster separately, demonstrating that the *CNGC* genes of these two species might evolve independently during evolution. Furthermore, intron numbers of groups II, IVa, and IVb differs between the two species, and group IVa *SlCNGC* (*SlCNGC15*) bore significantly more phase 1 type of introns than its *Arabidopsis* counterparts *AtCNGC19* and *AtCNGC20*. Additionally, size of introns of *SlCNGCs* is much larger than that of *AtCNGCs* (Supplementary Table S4, Supplementary Figure S9), suggesting tomato *CNGC* genes might be more highly expressed than and functionally discriminate from *Arabidopsis* CNGCs as suggested for other genes in *Arabidopsis* and rice (Ren et al., 2006).

Taken together, identification of tomato CNGC family establishes a basis to dissect the functions of CNGCs in crop plant species.

### Function of *SlCNGCs*

Cyclic nucleotide gated channels are involved in regulation of various biological processes such as growth and development, environmental stresses and plant defense responses (Chin et al., 2009; Ma, 2011). In the present study, we focused on dissection of the role of tomato CNGCs in disease resistance by conducting four levels of analyses, including prediction of *cis*-elements in promoter of *SlCNGC* genes, expression profiling of *SlCNGC* genes in response to diverse defense molecules and pathogens, pharmacological analyses on effect of cNMPs and Ca^2+^ channel activators/blockers on disease resistance and reverse genetics functional analyses of *SlCNGC* genes. All these analyses reveal that *SlCNGCs* are required for a wide range of disease resistances, and the effect is pathosystem-dependent. Regarding the expression profile, we found that treatment with BTH, a biological analog of SA, repressed the expression of *SlCNGC16*, the ortholog of *AtCNGC2*, and *SlCNGC17* and *SlCNGC18*, the orthologs of *AtCNGC4*. In contrast, treatments with both ETH and JA induced the expression of these group IVb CNGC genes in tomato (**Figure 3**). Our finding is similar to what have been observed in *Arabidopsis*, i.e., expression of both *AtCNGC2* and *AtCNGC4* are repressed by SA treatment while induced by MeJA treatment (Moeder et al., 2011). Moreover, we observed that expression of the three group IVb *SlCNGC* genes is distinct in response to the same pathogen inoculation, and all these genes respond distinctly to different types of pathogens. *SlCNGC16* is upregulated by inoculation with the biotrophic bacterial pathogens *Pst* DC3000 and *Xoo*, while is downregulated by inoculation with the necrotrophic fungal pathogen *Ss*, whereas the expression of *SlCNGC17* and *SlCNGC18* is just the opposite (**Figure 3**). Furthermore, VIGS analyses demonstrated that *SlCNGC17* and *SlCNGC18* are required for resistance to both *Ss* and *P. aphanidermatum*; while *SlCNGC16* is involved in resistance only to *Ss* but not to *P. aphanidermatum* (**Figure 4**). Our results suggest that group IVb *SlCNGC* genes play a role in both SA and JA/ETH signaling, however, which signaling pathway is activated and play a key role in defense is dependent on the pathosystem and therefrom the *CNGC* gene activated, as reported for group IVb *AtCNGCs* (Yoshioka et al., 2001; Balague et al., 2003; Genger et al., 2008; Moeder et al., 2011). Additionally, *AtCNGC11* and *AtCNGC12* are not essential to PTI. However, expression of *AtCNGC2* and *AtCNGC4* is suppressed after flg22 treatment (Moeder et al., 2011). Nevertheless, the role of *AtCNGC2* and *AtCNGC4* in PTI remains unclear. Besides, the respond of these genes to DAMPs is not yet analyzed. In this study, we found that silencing of group IVb *SlCNGC* genes reduced flg22- and AtPEP1-elicited hydrogen peroxide accumulation (**Figures 5A,B**), indicating that group IVb *SlCNGC* genes play a role in both PTI and DTI. Together with demonstration of the function of group IVb *SlCNGC* genes in tomato resistance to *Xoo*, *Ss* and *P. aphanidermatum*, our results extend the role of group IVb CNGCs in plant disease resistance.

As mentioned above, we found that tomato is responsive to AtPep1, which leads to accumulation of H_2_O_2_ (**Figure 5**). This suggests that a Pep1 receptor, most probably the receptor of the tomato ortholog of AtPep1, exists in tomato genome and is functional to recognize AtPep1 as well as its tomato homologs. This is conceivable considering the following findings. Firstly, it has been found that a Pep receptor could recognize several Peps sharing only moderate sequence identity. For example, the *Arabidopsis* Pep1 receptor PEPR1 can recognize other four Peps as well (Yamaguchi et al., 2006), which show only 35∼65% sequence identity to AtPep1 (Huffaker et al., 2006). Additionally, the signaling pathway downstream the recognition of Peps by PEPRs are conserved in plant species of different families. It has been reported that the downstream signaling initiated by recognition of AtPep1 by AtPEPR1 in *Arabidopsis* is also observed in the *AtPEPR1* transgenic tobacco when supplied with AtPep1 (Yamaguchi et al., 2006). Finally, an ortholog of AtPEP1 has been identified in tomato. This ortholog is 39% identical to sequence of AtPep1. Moreover, it is involved in resistance to *Pythium dissotocum* through signaling pathways mediated by jasmonic acid/ethylene (JA/ET; Trivilin et al., 2014), as reported for AtPep1 (Huffaker et al., 2006).

It is notable that in the pharmacological study, supply with putative CNGC activators cAMP and cGMP, thus expected to activate CNGCs, enhance tomato resistance to *Ss* (Supplementary Figure S1), which seems to be contradictory to the result from the CNGC-VIGS analyses that silencing of group IVb *SlCNGC* genes, thus expected to decrease the activity of these CNGCs, enhance the same resistance (**Figures 4D,E**). One explanation for this contradiction is that cAMP and cGMP generally activate the whole CNGC family in tomato, and some of them play a role opposite to group IVb CNGCs in regulating this resistance. The CNGC gene-dependent function has been observed in this study. Members of *SlCNGC* gene family respond in expression variously to the same pathogen inoculation (**Figure 3**). Furthermore, VIGS analysis demonstrates that *SlCNGC17* and *SlCNGC18*, but not *SlCNGC16* play a role in resistance to *P. aphanidermatum* (**Figures 4F,G**); while *SlCNGC16* and *SlCNGC18*, but not *SlCNGC17* function in flg22-elicited hydrogen peroxide accumulation (**Figure 5A**). Another possibility is the pleiotropism of cAMP and cGMP. They activate not only CNGCs but also other targets, which function opposite to the CNGCs in the resistance. For instance, beside CNGCs, cAMP activates cAMP-dependent protein kinases (PKAs) and guanine nucleotide exchange factors (GEFs) for small GTPase as well (Bos, 2003). It is possible that some PKAs and/or GEFs may regulate resistance in contrast to CNGCs. Similar observation has been reported for *P. patens CNGCb* gene. When an abrupt, continuous heat treatment at 34 or 38°C was applied, the 24°C-grown *P. patens CNGCb* mutant does not show reduced Ca^2+^ influx and decreased cytosolic Ca^2+^, in contrast, they display a stronger Ca^2+^ influx and elevated cytoplasmic Ca^2+^ concentration. This might be attributed to the promotive effect of *PpCNGCb* mutation on opening of other related Ca^2+^ channels (Finka et al., 2012). Interestingly, tomato CNGC16, CNGC17, and CNGC18 are the orthologs of *Arabidopsis* CNGC2 and CNGC4 (**Figure 2**), which are the orthologs of *PpCNGCb* (Finka et al., 2012). Therefore, whether similar mechanism occurs for tomato group IVb CNGCs deserves to be further analyzed.

### Functional Mechanisms of *SlCNGCs*

The electrophysiological studies have suggested that some CNGCs such as AtCNGC18 are Ca^2+^-permeable channels (Gao et al., 2014; Zhou et al., 2014), thus we examined the effect of *SlCNGCs* on expression of the Ca^2+^ signaling related genes. The results demonstrated that silencing of group IVb *SlCNGC* genes reduced the expression of *SlCaM6*, *SlCDPK2*, *SlCDPK11,* and *SlCAMTA3* but differentially altered the expression pattern of *SlCaM2*, i.e., silencing of *SlCNGC16* and *SlCNGC18* up-regulated while silencing of *SlCNGC17* down-regulated the expression of *SlCaM2* (**Figure 6**). Our previous results reveal that silencing of *SlCaM2* and *SlCaM6* enhances expression of *SlCNGC17* but reduces expression of the *SlCNGC18* and both reduce resistance to *P. aphanidermatum* (Zhao et al., 2013). Collectively, these results indicate the complexity of interactions between CNGC and CaM during disease resistance. In addition, *CNGCs* regulate disease resistance through promoting *SlCDPK2*, *SlCDPK11,* and *SlCAMTA3*. Besides, silencing of group IVb *SlCNGC* genes obviously reduces PAMP- and DAMP-triggered hydrogen peroxide accumulation (**Figure 5**), demonstrating that these *CNGCs* affect PTI and its amplification through regulating PAMP- and DAMP-triggered hydrogen peroxide accumulation. More detailed functional mechanisms of *SlCNGCs* remain further dissection.

## Conclusion

Cyclic nucleotide gated channels are multifunctional and have been supposed to be calcium conducting channels. Nevertheless, genome-wide identification of plant *CNGC* gene family has been conducted only in four flowering plant species *Arabidopsis*, rice, pear and *Populus trichocarpa*, a moss *P. patens* and some alga species. Furthermore, systemic functional analysis of plant *CNGC* genes has not been well performed except in *Arabidopsis*. In this study, we identified *CNGC* gene family in the economically important crop tomato (*Solanum lycopersicum* L.) and analyzed function of the group IV *SlCNGC* genes in disease resistance, representing the first genome-wide identification and functional analysis of *CNGC* gene family in dicotyldons crop species. Importantly, the tomato CNGC gene family is not only identified through complex bioinformatics analyses but also confirmed by PCR cloning and sequencing. We correct four *CNGC* loci that were misannotated at database. We demonstrate that gene structure, domain composition and phylogenetic relationship of the *SlCNGC* gene family are group-specific. The tomato CNGC gene family contains less members, carries different exon/intron gene structures but own similar groups when compared with *Arabidopsis CNGCs*. We conduct comprehensive expression analyses and reveal that *SlCNGC* genes are highly and widely responsive to diverse stimuli in a gene-dependent manner. Employing pharmacological assays and VIGS technique, we further unravel the function of the group IVb *SlCNGC* genes in disease resistance. Silencing of these *SlCNGC* genes significantly enhances resistance to fungal pathogens *P. aphanidermatum* and *S. sclerotiorum*, strongly reduces resistance to viral pathogen *Tobacco rattle virus*, while attenuates PAMP- and DAMP-triggered immunity as shown by obvious decrease of flg22- and AtPEP1-elicited hydrogen peroxide accumulation in *SlCNGC*-silenced plants. Finally, this work indicates that the group IVb *SlCNGC* genes regulate a wide range of resistance to diverse pathogens in tomato probably via affecting Ca^2+^ signaling including *SlCaMs*, *SlCDPKs,* and *SlCAMTA3*. Collectively, this work identifies *CNGC* gene family in tomato genome, provides some insights into functions of tomato CNGCs and thus makes a platform to further elucidate the functions of plant CNGCs.

## Author Contributions

MS and Y-P X conducted the bioinformatics, phylogenetic and pharmacological analyses. MS, WL and J-PW carried out the gene expression, ROS detection and VIGS analysis. MS and Y-P X designed and analyzed all statistical data. X-Z C conceived of the study, and participated in its design and coordination. X-Z C and MS prepared the manuscript.

## Footnotes

1 http://www.arabidopsis.org/

2 http://solgenomics.net/

3 http://pfam.sanger.ac.uk/

4 http://smart.embl-heidelberg.de/

5 http://www.ncbi.nlm.nih.gov/cdd

6 http://gsds.cbi.pku.edu.cn/

7 http://www.dna.affrc.go.jp/PLACE/signalscan.html

8 http://www.arabidopsis.org/

9 http://solgenomics.net/

10 http://pfam.sanger.ac.uk/

11 http://smart.embl-heidelberg.de/

12 http://www.ncbi.nlm.nih.gov/Structure/cdd/wrpsb.cgi/
