# Supplementary material for: Cyclic nucleotide gated channel gene family in tomato: genome-wide identification and functional analyses in disease resistance
Source: Front Plant Sci. 2015 May 5;6:303. doi: 10.3389/fpls.2015.00303 (PMC4419669; doi:10.3389/fpls.2015.00303)
Supplement: Supplementary file 1 [file Data_Sheet_1.PDF]

Supplementary Material

Cyclic nucleotide gated channel gene family in tomato: genome-wide identification and functional analyses in disease resistance

Mumtaz Ali Saand[1], You-Ping Xu[2], Wen Li[1], Ji-Peng Wang[1], and Xin-Zhong Cai[1*]

[1] Institute of Biotechnology, College of Agriculture and Biotechnology, Zhejiang University, Hangzhou, China

[2] Centre of Analysis and Measurement, Zhejiang University, Hangzhou, China

[*]Correspondence:

Xin-Zhong Cai

Institute of Biotechnology, College of Agriculture and Biotechnology, Zhejiang University, 866 Yu Hang Tang Road, Hangzhou 310058, China;

Tel: +86-571-88982936

Fax: +86-571-88982936

E-mail: xzhcai@zju.edu.cn

Supplementary Tables

Table S1 | Primers used in this study.

Name of primer	Sequence (5'→ 3')	Anticipated PCR product (bp)
**SlCNGC gene cloning**		
SlCNGC5-F	ATGAATCACCGACAAGAGGAGTTCT	
SlCNGC5-R	TTACTTATCTTCAGCAGTGAAATCT	2127
SlCNGC6-F	ATGAATCACCGGCAAGAGAA	
SlCNGC6-R	TTACTTATCTTCAGCAGTAA	2127
SlCNGC15-F	ATGCCGATGTTATCACCATCAT	
SlCNGC15-R	TTAGCGTTGGGGGCTGGAAGAG	2292
**qRT-PCR analysis**		
SlCNGC1-F	GAACACTGCAAGTCAGAAGA	
SlCNGC1-R	TCACTTAGGAAAGGGGTGCA	156
SlCNGC3-F	TTCTGGTACATAGAAACACA	
SlCNGC3-R	TTCATTACTGTCCCTGAAGT	58
SlCNGC5-F	ACTAAAGTTGTAATAGCTTC	
SlCNGC5-R	AGCTTTCTTCCACAAAACCA	158
SlCNGC6-F	GGGCAGATTTGGAGTGTTCT	
SlCNGC6-R	AACTTTTGACCAAAAGGCAA	119
SlCNGC7-F	GTTCGACTGTGGTTCCAAGT	
SlCNGC7-R	TGAAGCCACATGTATTTGT	125
SlCNGC8-F	ATTAGTCTTATTGCTACATA	
SlCNGC8-R	GTGTCTTCTACTTATCACGG	136
SlCNGC9-F	AGTCGCAGATTATTGGAGGA	
SlCNGC9-R	TGTCACTAGTCCCTCAGATC	185
SlCNGC10-F	GTGGCTCAACGATGGCAAAT	
SlCNGC10-R	TCAAAACTGCAGGCGTGTC	256
SlCNGC12-F	AGGAATAGCAGACAGTTCGA	
SlCNGC12-R	CTTCCTCCAGGACTCATGGT	114
SlCNGC14-F	CTCAACCGCTTCAAGAATTC	
SlCNGC14-R	TAACCCAATAGATTGTCGGT	203
SlCNGC15-F	CATCATATCCACAGTCTGAT	
SlCNGC15-R	CTCATTTGAACCAATGAAGT	181
SlCNGC16-F	ACCTGATAACAGCGACGACA	
SlCNGC16-R	TGAATTGGAACTAGAGAAGA	168
SlCNGC17-F	CTGAAGATGAAGATGAAGAT	
SlCNGC17-R	GTTTCTGTCATCACGACTA	122

SlCNGC18-F	TTATAGTGATCGAAGTGACG	
SlCNGC18-R	AAGAAGTCTGTCAATCCTCC	144
TRV2 2b-F	ATGCACGAATTACTTAGGAAG	
TRV2 2b-R	GGTAACCTTACTCACAGAAT	303
TRV1 Rep-F	ATCTCAAGTTGATTTGAGGTT	
TRV1 Rep-R	TGATCTCTTTGCTTACATCGT	461
SlCaM2-F	CTGATGAAGAAGTCGATGAGATG	
SlCaM2-R	AGACAAGAGCCTACCCAATGA	186
SlCaM6-F	ATGAGATGATCCGAGAGG	
SlCaM6-R	AGGCCACTAATATACTTGAACC	191
SlCDPK2-F	ATAAGATATTGAACCGTTGG	
SlCDPK2-R	GTCATCCGGCACCCGAGCC	148
SlCDPK11-F	CTACTCTCAGCAACAGCATG	
SlCDPK11-R	TGAACTAGAGGAAAAACCCT	129
SlCAMTA3-F	TGATGCATGAACAAGGAAAC	
SlCAMTA3-R	TCAACTTCTGATCCATGGAC	156
**VIGS analysis**		
SlCNGC16-F	gcgaattcATATCTTGCCTCATTCTTCA	
SlCNGC16-R	ttggatccACGCCAACTTGAGTACATGC	299
SlCNGC17-F	gcgaattcGACGTTCTTGTCTAAAATCT	
SlCNGC17-R	ttggatccGTACTCGTCTTCTTCTAGCA	312
SlCNGC18-F	gcgaattcTTATAGTGATCGAAGTGACG	
SlCNGC18-R	ttggatccAAGAAGTCTGTCAATCCTCC	144

Table S2 | List of the truncated *SlCNGC* genes identified in this study.

Gene locus	Protein size (aa)	Mol Wt (kDa)	pI	Intron	Chromosome/position	Domain organization			
						Pfam		SMART	CDD
						Significant	Insignificant		
Solyc06g010190.1.1	404	4.634	9.46	8	ch06 5308138-5305190	—	—	2TMD	
Solyc06g010180.1.1	127	1.475	8.70	1	ch06 5300684-300221	—	CNBD	cNMP	CAP_ED
Solyc02g086990.2.1	445	5.171	8.42	5	ch02 44128131-44124213	ITP	Helix-turn-helix 17*	4TMD	Adenosine-deaminase*

Abbreviations used in table: ITP (Ion transport protein), CNBD (Cyclic nucleotide-binding domain), cNMP (Cyclic nucleotide-monophosphate binding domain), TMD (Transmembrane domain), CAP_ED (Effector domain of CAP family).
The asterisks showed in table indicate the insignificant or incomplete domains.

Table S3 | Domain organization of AtCNGCs.

Group	Gene symbol	Gene locus	Protein size (aa)	Intron	Domain organization			
					Pfam		SMART	CDD
					Significant	Insignificant		
I	AtCNGC11	At2g46440	621	7	ITP, CNBD	—	cNMP, 5TMD	CAP_ED
	AtCNGC12	At2g46450	649	8	ITP, CNBD	—	cNMP, 5TMD	ITP, CAP_ED
	AtCNGC3	At2g46430	706	7	ITP	CNBD	cNMP, 5TMD	ITP, CAP_ED
	AtCNGC10	At1g01340	711	8	ITP, CNBD	—	cNMP, 5TMD	ITP, CAP_ED
	AtCNGC13	At4g01010	696	6	ITP, CNBD	—	cNMP, 5TMD	CAP_ED
	AtCNGC1	At5g53130	716	7	ITP, CNBD	IQ CaM BM*	cNMP, 5TMD	ITP, CAP_ED
II	AtCNGC07	At1g15990	709	4	ITP, CNBD	—	cNMP, 4TMD	ITP, CAP_ED
	AtCNGC08	At1g19780	753	5	ITP, CNBD	IQ CaM BM*, TMD of TG (PBP1) *	cNMP, 5TMD	CAP_ED
	AtCNGC05	At5g57940	717	7	ITP, CNBD	IQ CaM BM*	cNMP, 3TMD	ITP, CAP_ED
	AtCNGC06	At2g23980	747	7	ITP, CNBD	IQ CaM BM*	cNMP, 6TMD	ITP, CAP_ED
	AtCNGC09	At4g30560	707	6	ITP, CNBD	IQ CaM BM*	cNMP, 5TMD	ITP, CAP_ED
III	AtCNGC15	At2g28260	678	5	ITP, CNBD	IQ CaM BM*	cNMP, 5TMD	ITP, CAP_ED
	AtCNGC17	At4g30360	720	5	ITP, CNBD	IQ CaM BM*	cNMP, 6TMD	ITP, CAP_ED
	AtCNGC14	At2g24610	726	6	ITP, CNBD	—	cNMP, 6TMD	ITP, CAP_ED
	AtCNGC18	At5g14870	706	5	CNBD	—	cNMP, 7TMD	CAP_ED, DUF (4414) *
	AtCNGC16	At3g48010	705	6	ITP, CNBD	—	cNMP, 4TMD	ITP, CAP_ED
IVa	AtCNGC19	At3g17690	729	9	ITP	—	cNMP, 5TMD	CAP_ED
	AtCNGC20	At3g17700	764	10	ITP	—	cNMP, 6TMD	ITP, CAP_ED
IVb	AtCNGC2	At5g15410	725	7	CNBD	ITP, REP (GP41) *, TMEMspv1-c74-12*	cNMP, 7TMD	CAP_ED
	AtCNGC4	At5g54250	694	7	CNBD	ITP	cNMP, 7TMD,	ITP, CAP_ED, NP*

Abbreviations used in table: ITP (Ion transport protein), cNBD (Cyclic nucleotide-binding domain), IQ CaM BM (IQ Calmodulin-binding motif), TMD of TG (Transmembrane domain of transglycosylase), PBP1 (Penicillin-binding protein 1), REP (Retroviral envelop protein), GP41 (Glycoprotein 41), TMEMspv1 (Transmembrane protein spiroplasma virus 1), cNMP (Cyclic nucleotide-monophosphate

binding domain), TMD (Transmembrane domain), CAP_ED (Effector domain of CAP family), DUF (Domain of unknown function), NP (Nucleoplasmin). The asterisks showed in table indicate the insignificant or incomplete domains.

Table S4 | The introns of the *AtCNGC* and *SlCNGC* genes.

Group	CNGC	Locus number	Gene size (Kb)	Intron number			Total number of introns
				Phase 2	Phase 1	Phase 0	
I	AtCNGC1	At5g53130	3.200	3	0	4	7
	AtCNGC10	At1g01340	3.502	4	0	4	8
	AtCNGC11	At2g46440	2.547	3	0	4	7
	AtCNGC12	At2g46450	3.725	3	0	5	8
	AtCNGC13	At4g01010	2.674	3	0	3	6
	AtCNGC3	At2g46430	2.802	3	0	4	7
	SlCNGC1	Solyc01g095770.2.1	3.610	4	0	4	8
	SlCNGC2	Solyc05g050380.2.1	4.369	3	0	4	7
	SlCNGC3	Solyc05g050350.1.1	3.935	3	0	4	7
	SlCNGC4	Solyc05g050360.2.1	5.219	3	0	4	7
	SlCNGC5	Corrected Solyc06g051920.2.1	4.386	2	2	4	8
	SlCNGC6	Corrected (Solyc03g007260.2.1 + Solyc03g007250.1.1)	2.127	2	0	4	6
II	AtCNGC05	At5g57940	3.773	3	0	4	7
	AtCNGC06	At2g23980	3.189	3	0	4	7
	AtCNGC07	At1g15990	2.469	1	0	3	4
	AtCNGC08	At1g19780	2.694	2	0	3	5
	AtCNGC09	At4g30560	3.199	3	0	3	6
	SlCNGC7	Solyc07g005590.2.1	5.889	2	0	4	6
	SlCNGC8	Solyc12g010010.1.1	6.640	1	0	4	5
	SlCNGC9	Solyc03g116850.2.1	4.767	2	0	4	6
III	AtCNGC14	At2g24610	3.247	2	0	4	6
	AtCNGC15	At2g28260	2.465	2	0	3	5
	AtCNGC16	At3g48010	2.694	2	0	4	6
	AtCNGC17	At4g30360	2.720	2	0	3	5
	AtCNGC18	At5g14870	2.630	1	0	4	5
	SlCNGC10	Solyc11g069580.1.1	4.150	2	0	4	6
	SlCNGC11	Solyc09g007840.2.1	2.952	2	0	4	6
	SlCNGC12	Solyc07g006510.2.1	8.451	2	0	4	6
	SlCNGC13	Solyc08g069140.2.1	3.654	2	0	4	6
	SlCNGC14	Solyc03g114110.2.1	5.216	1	0	4	5
IVa	AtCNGC19	At3g17690	2.958	2	1	6	9
	AtCNGC20	At3g17700	3.376	2	1	7	10
	SlCNGC15	Corrected Solyc03g098210.2.1	13.667	1	7	4	12
IVb	AtCNGC2	At5g15410	3.304	2	0	5	7
	AtCNGC4	At5g54250	4.288	2	0	5	7
	SlCNGC16	Solyc02g088560.2.1	3.450	2	0	5	7
	SlCNGC17	Solyc10g006800.2.1	6.146	3	0	5	8
	SlCNGC18	Solyc12g005400.1.1	5.920	2	0	5	7

Supplementary Figures

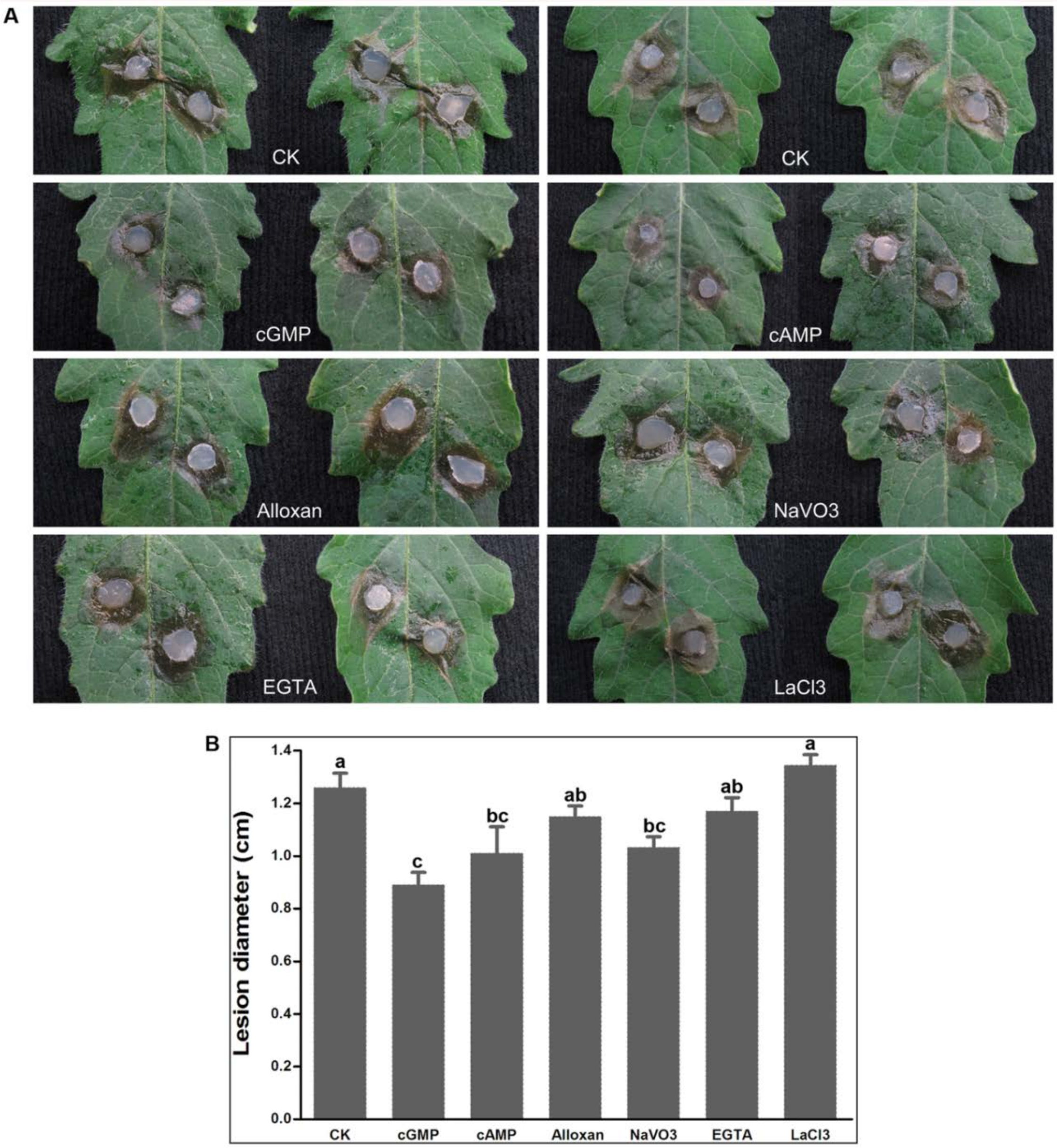

Figure S1 | Effect of Ca^{2+} and Ca^{2+} channel effectors on resistance against *Sclerotinia sclerotiorum* in tomato. (A) The necrotic disease symptoms caused by *Sclerotinia sclerotiorum* inoculation in leaves infiltrated with sterilized water (CK), cGMP (1 mM), cAMP (100 μM), $NaVO_3$ (50 μM), alloxan (1 mM), EGTA (1 mM) and $LaCl_3$ (1 mM) respectively. The photographs were taken at 44 hpi. **(B)** Statistical analysis of the lesion diameter measured for all inoculated leaves. The small letters over bar denote significant differences between CK and chemical treatments ($p<0.05$, DMRT). The experiments were conducted three times, the mean ± SE represents the three independently repeats.

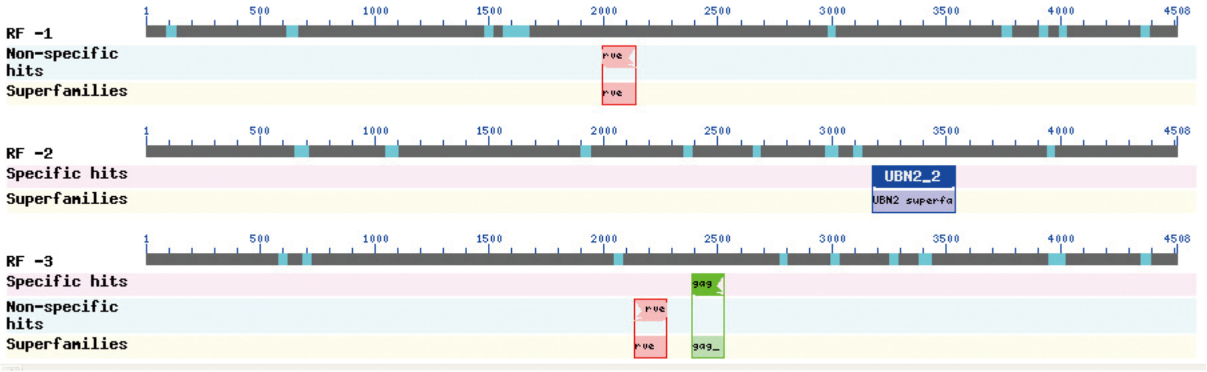

Figure S2 | Domain composition of the 4506 bp insertion between sequences Solyc06g010180.1.1 and Solyc06g010190.1.1. The domain composition was analyzed by NCBI-CDD. The result indicated the possible retrotransposon-mediated loss of a functional CNGC protein by 4506 bp insertion between sequences Solyc06g010180.1.1 and Solyc06g010190.1.1.

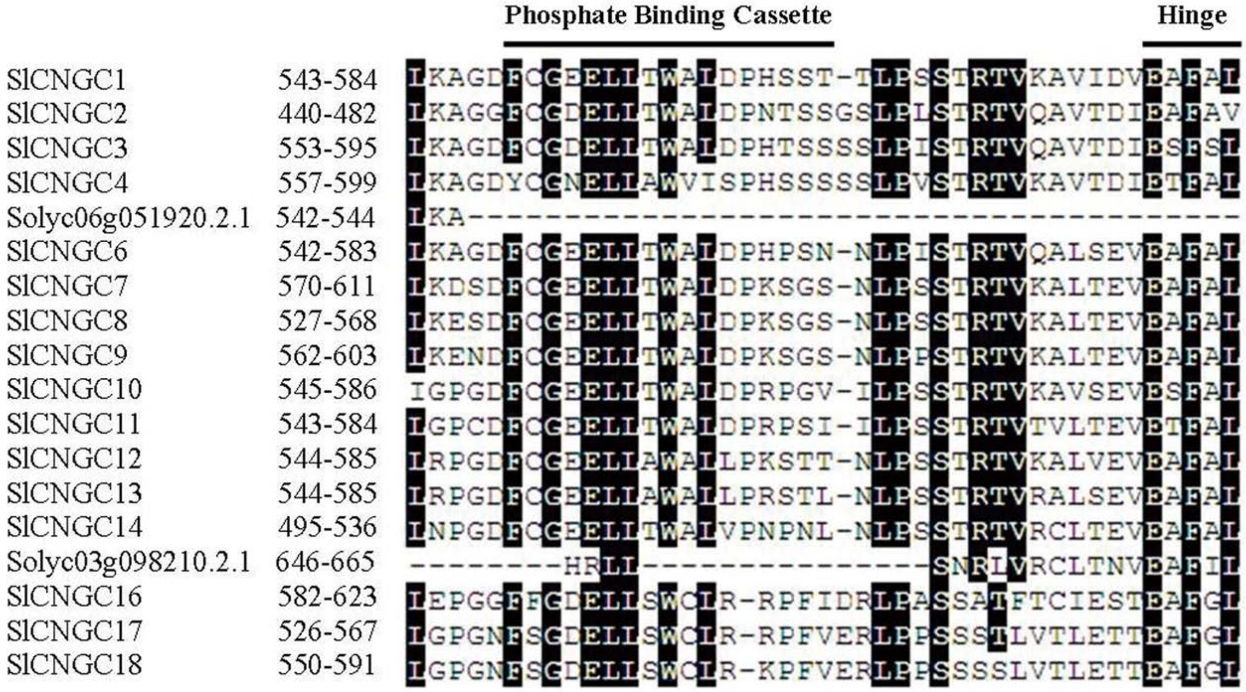

Figure S3 | The CNGC-specific motif region spanning the PBC and hinge region within CNB domain of 18 SlCNGCs. The names and residue positions from the N-termini of SlCNGCs are indicated to the left of the motif region. The phosphate biding cassette (PBC) and hinge region are indicated by black lines above of sequences. The deletion of both PBC and hinge region in Solyc06g051920.2.1 and only PBC in Solyc03g098210.2.1 is indicated as ″-″ in alignment. Residues in white highlighted in black indicate >80% conservation among SlCNGCs. The SlCNGC-specific motifs were aligned by ClustalW and generated by MEGA5 program.

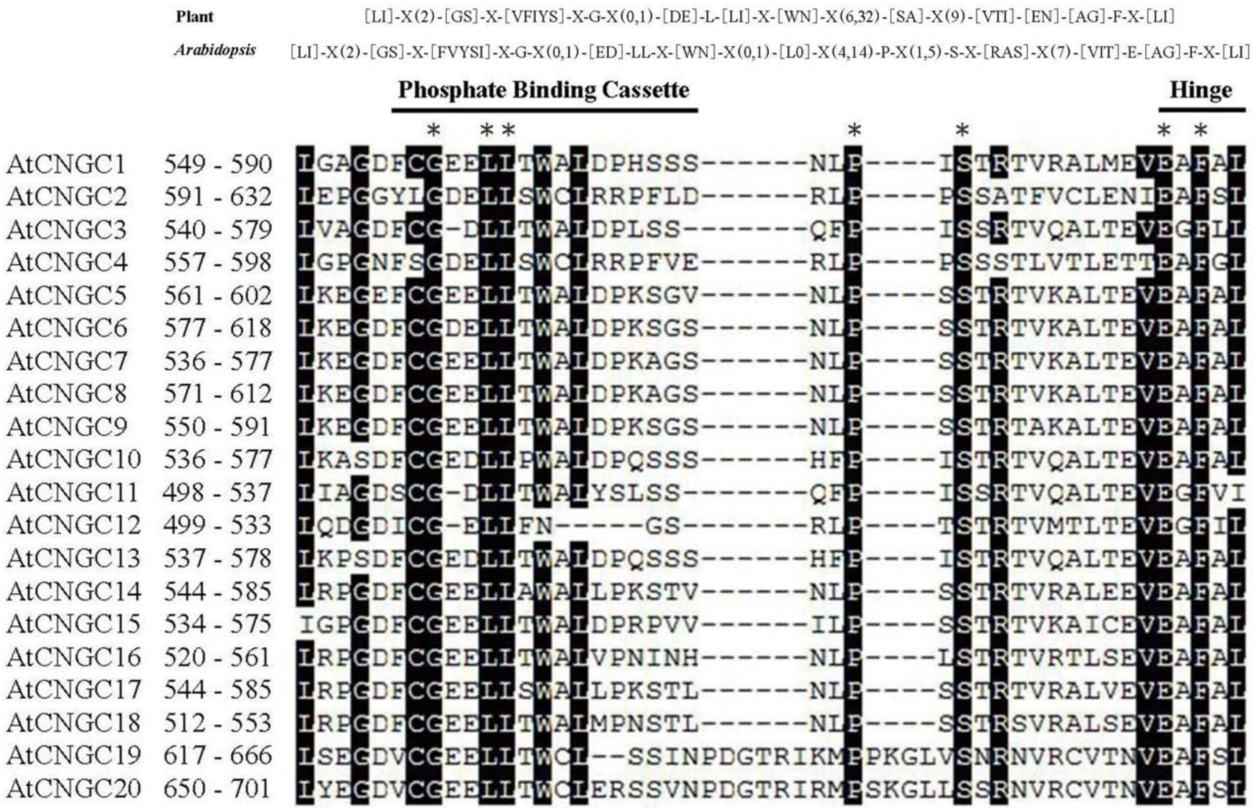

Figure S4 | The CNGC-specific motif spanning the phosphate binding cassette (PBC) and hinge region within CNB domain of 20 AtCNGCs. The CNGC-specific motifs for all plant species (Zelman et al., 2012) or Arabidopsis only are shown at top of alignment respectively. The square brackets "[ ]" indicate the amino acids allowed in this position of motif, "X" represents any amino acid, while round brackets "()" denote the number of amino acids. The names and residue positions from the N-termini of AtCNGCs are indicated to the left of the motif. The PBC and hinge region are indicated by black lines above sequences. Residues in white highlighted in black indicate >90% conservation among AtCNGCs. The asterisks above the alignment indicate 100% conservation among all Arabidopsis CNGCs. The AtCNGC-specific motifs were aligned by ClustalW and generated by MEGA5 program.

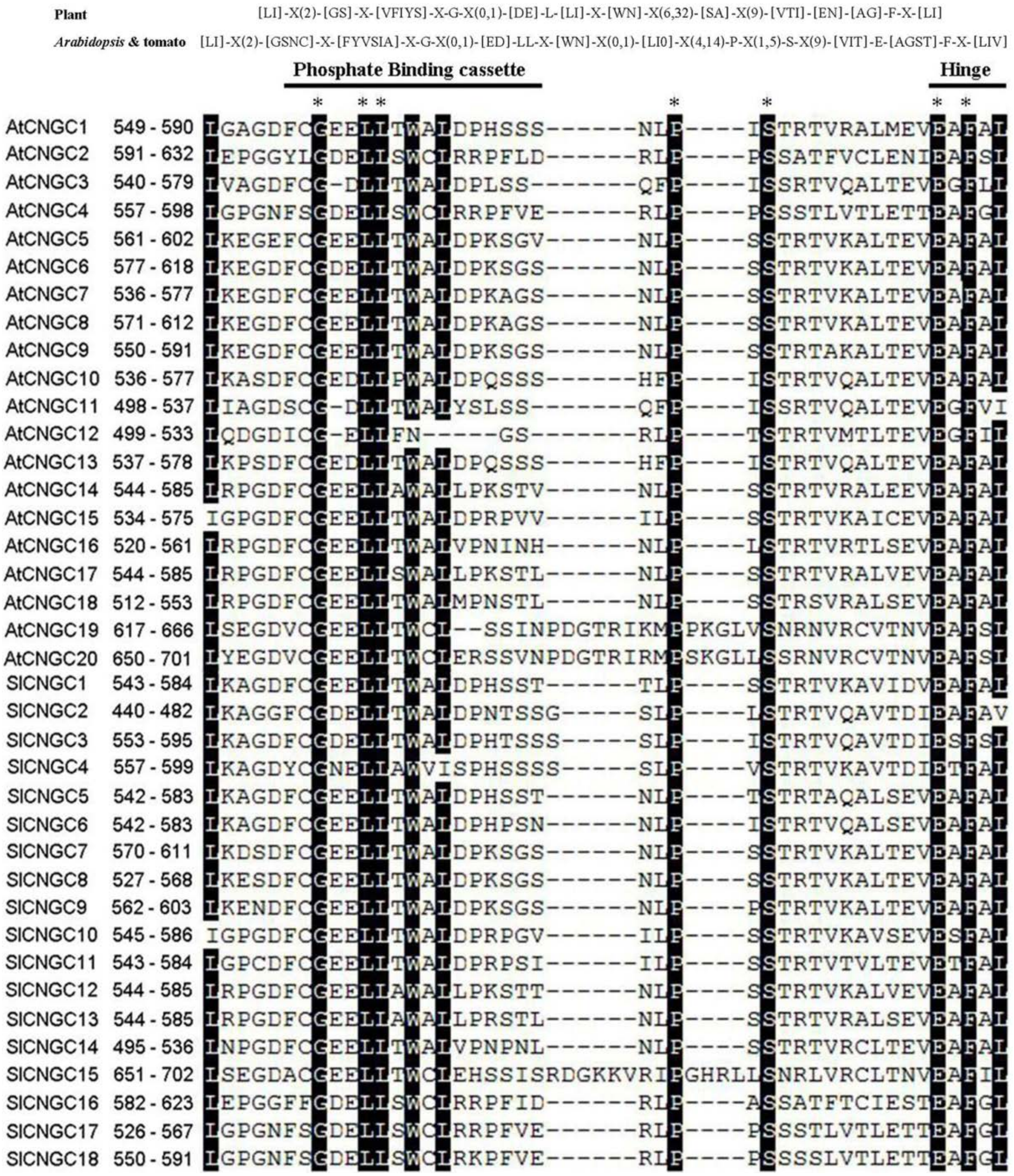

Figure S5 | The CNGC-specific motif region spanning the PBC and hinge region within CNB domain of 20 AtCNGCs and 18 SlCNGCs. The CNGC-specific motifs of all plant species (Zelman et al., 2012) and Arabidopsis and tomato only are shown at top of alignment respectively. The square brackets "[ ]" indicate the amino acids allowed in this position, "X" represents any amino acid, while round brackets "()" denote the number of amino acids. The names and residue positions from the N-termini of AtCNGCs and SlCNGCs are indicated to the left of the motif. The phosphate biding cassette (PBC) and hinge region are indicated by black lines above sequences. Residues in white highlighted in black indicate >90% conservation among AtCNGCs and SlCNGCs. The asterisks above the alignment indicate 100% conservation among all *Arabidopsis* and tomato CNGCs. The CNGC-specific motifs were aligned by ClustalW and generated by MEGA5 program.

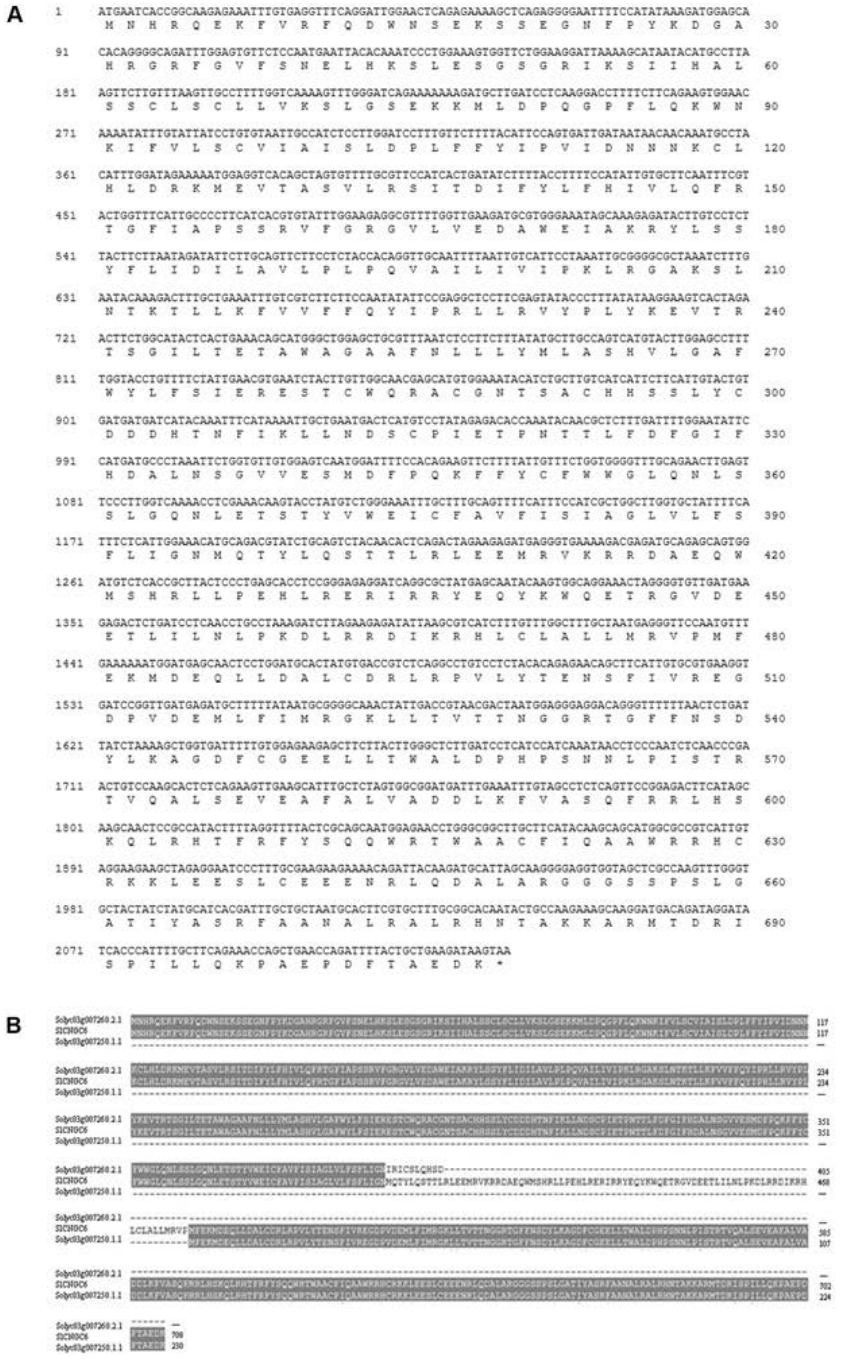

Figure S6 | *SlCNGC6* cloned in this study. (A) The coding nucleotide sequence and deduced protein sequence of *SlCNGC6* cloned from tomato cv. Heinz 1706 (GenBank accession no. KJ499457). **(B)** The alignment among SlCNGC6 and the two truncated sequences from Solyc03g007260.2.1 and Solyc03g007250.1.1 deposited in SGN. The numbers at left and right denote the position of nucleotide and protein sequences respectively. The alignment was generated by GeneDoc program.

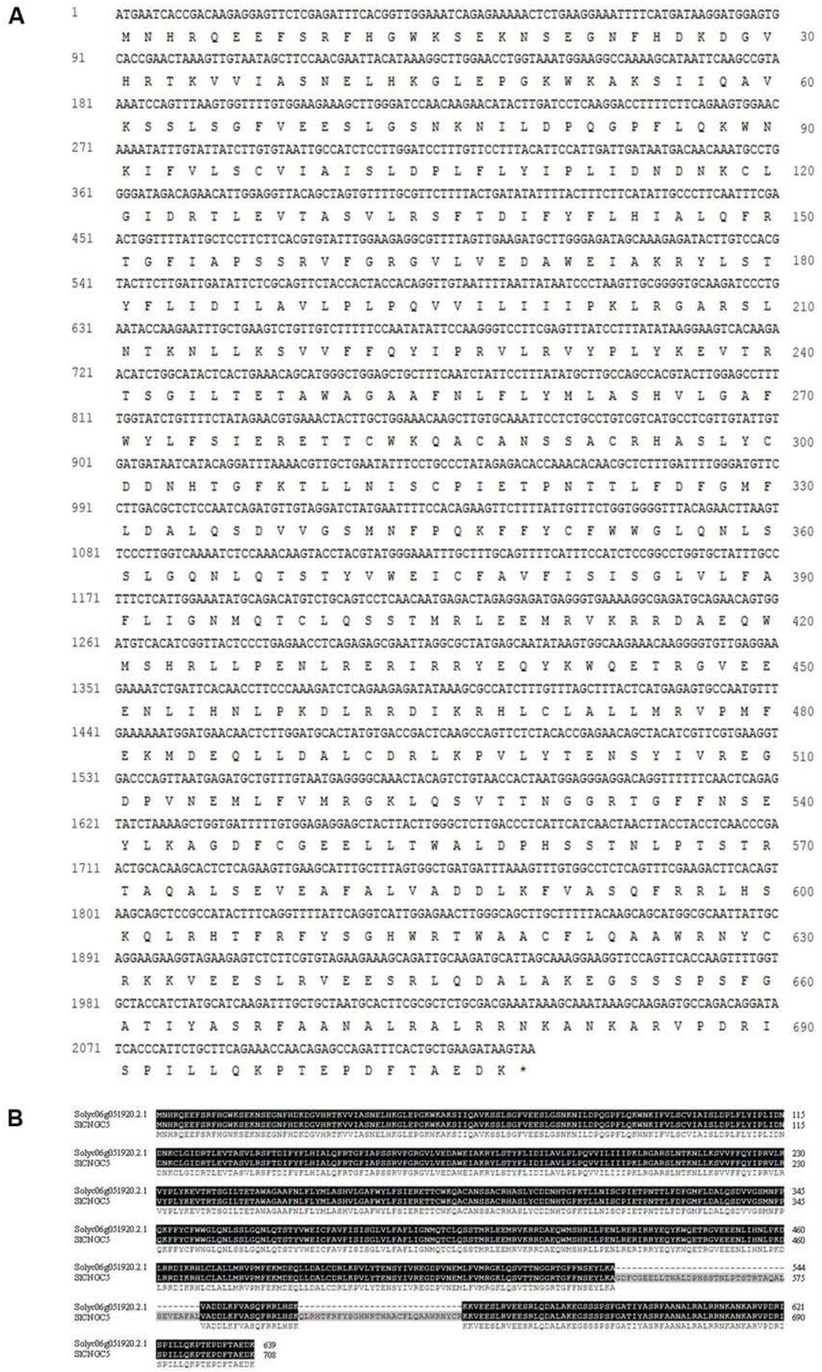

Figure S7 | *SlCNGC5* cloned in this study. (A) The coding nucleotide sequence and deduced protein sequence of *SlCNGC5* cloned from tomato cv. Heinz 1706 (GenBank accession no. KJ499456). **(B)** The alignment among SlCNGC5 and the truncated sequence from Solyc06g051920.2.1 deposited in SGN. The numbers at left and right denote the position of nucleotide and protein sequences respectively. The alignment was generated by GeneDoc program.

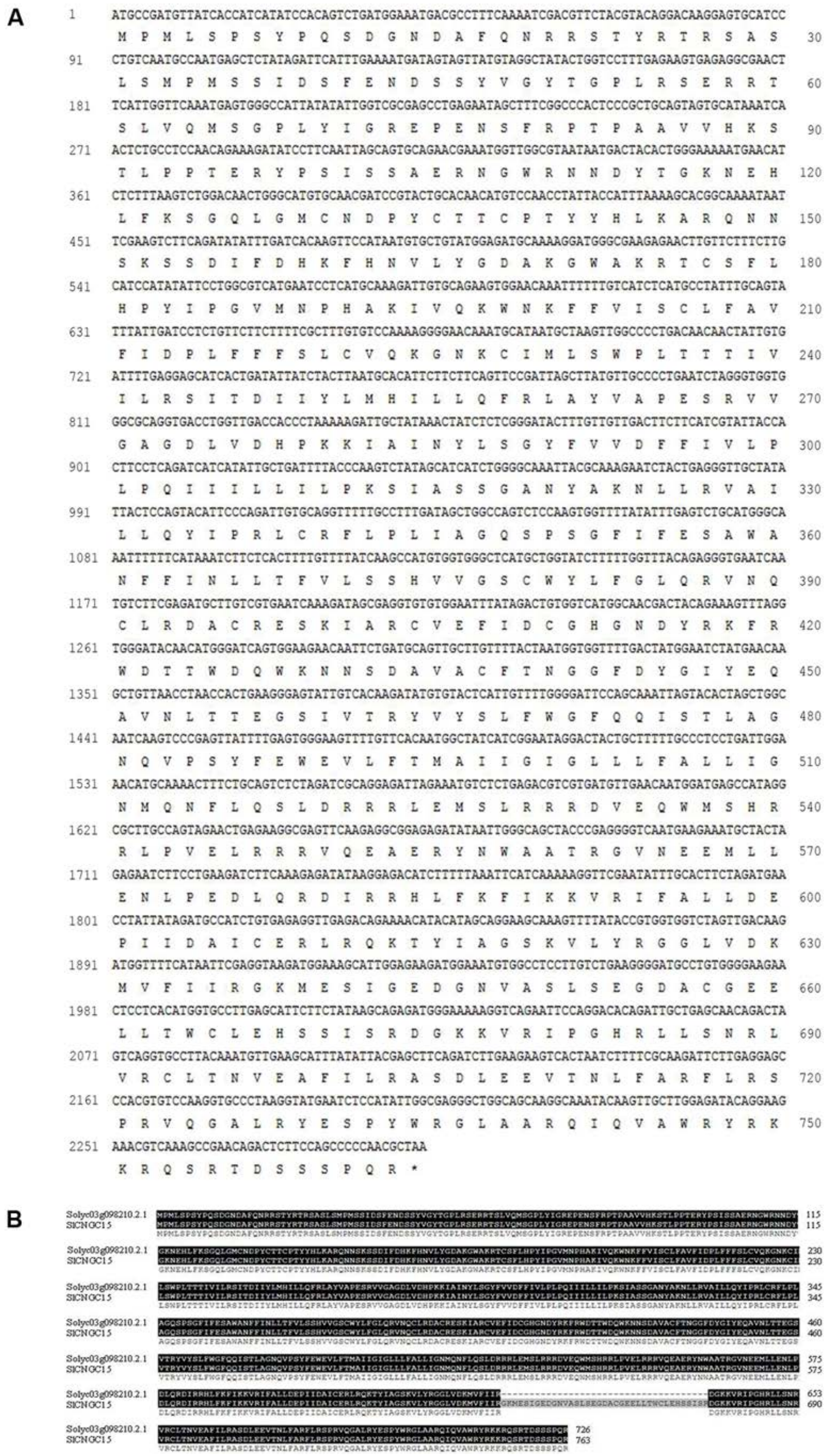

Figure S8 | *SlCNGC15* cloned in this study. (A) The coding nucleotide sequence and deduced protein sequence of *SlCNGC15* cloned from tomato cv. Heinz 1706 (GenBank accession no. KJ499458). **(B)** The alignment among SlCNGC15 and the truncated sequence from Solyc03g098210.2.1 deposited in SGN. The numbers at left and right denote the position of nucleotide and protein sequences respectively. The alignment was generated by GeneDoc program.

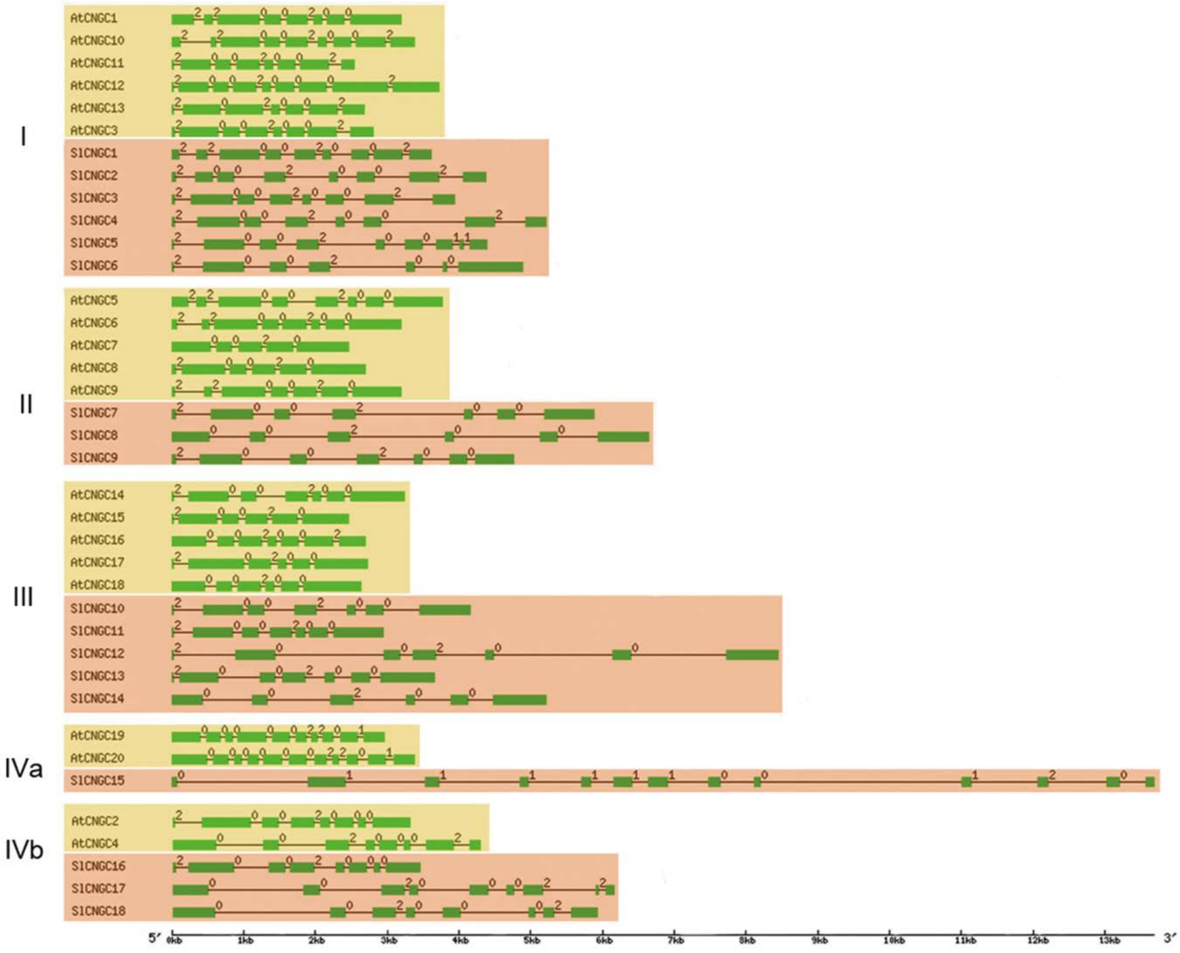

Figure S9 | The predicted exon/intron struture of the *AtCNGC* and *SlCNGC* genes. Group numbers were indicated in the left. *AtCNGCs* and *SlCNGCs* were highlighted in yellow and orange respectively. Green boxes and black lines represent exons and introns respectively. The numbers 0, 1 and 2 indicate the intron phases.

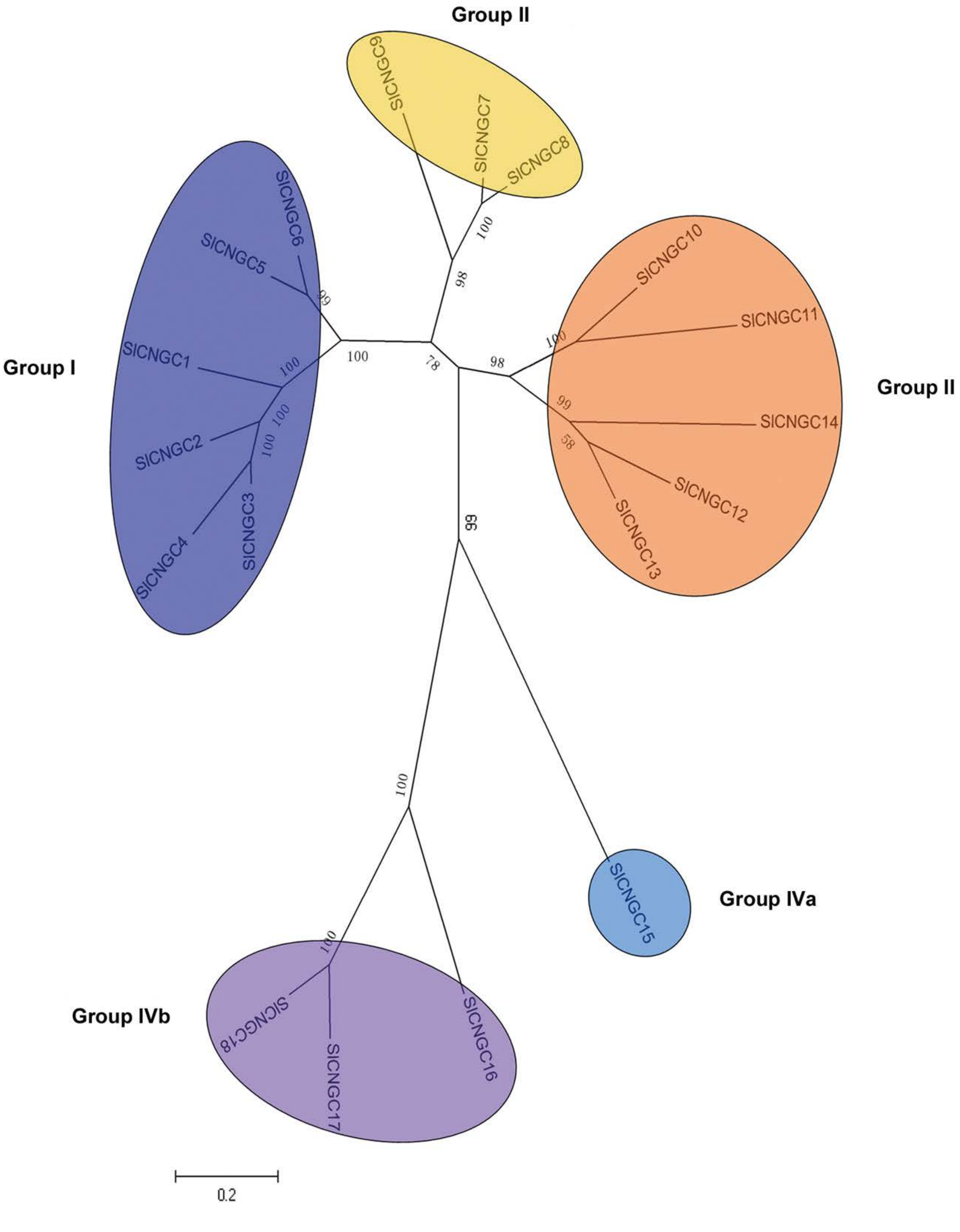

Figure S10 | Phylogentic tree of SlCNGC proteins. The tree was created using Clustalx program by maximum liklihood (ML) method with 1000 bootstrap in MEGA. The circles with different colours represent tomato CNGC genes of diverse groups.

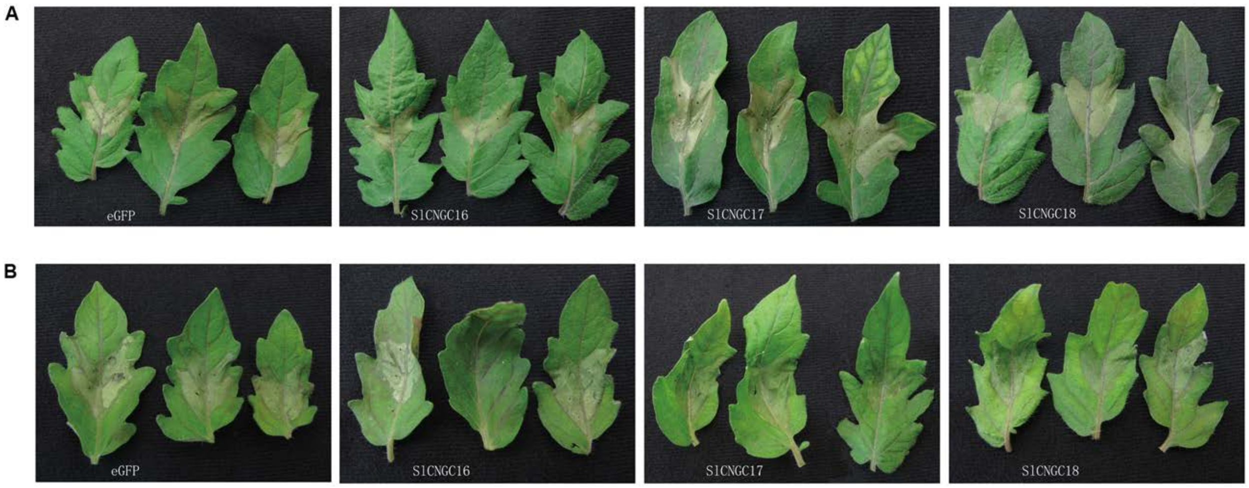

Figure S11 | Hypersensitive response (HR) symptoms in group IVb *SlCNGC* gene-silenced plants inoculated with bacterial pathogens *Xoo* and *Pst* DC3000. Plants infiltrated with *Agrobacterium* suspensions carrying an eGFP control vector were served as control plants. Photographs were taken at 14 h post *Xoo* inoculation **(A)** and 48 h post *Pst* DC3000 inoculation **(B)**.
